# Microevolution from shock to adaptation revealed strategies improving ethanol tolerance and production in *Thermoanaerobacter*

**DOI:** 10.1186/1754-6834-6-103

**Published:** 2013-07-22

**Authors:** Lu Lin, Yuetong Ji, Qichao Tu, Ranran Huang, Lin Teng, Xiaowei Zeng, Houhui Song, Kun Wang, Qian Zhou, Yifei Li, Qiu Cui, Zhili He, Jizhong Zhou, Jian Xu

**Affiliations:** 1 BioEnergy Genome Center, CAS Key Laboratory of Biofuels and Shandong Key Laboratory of Energy Genetics, Qingdao Institute of Bioenergy and Bioprocess Technology, Chinese Academy of Sciences, Qingdao, Shandong, P. R. China; 2 Institute for Environmental Genomics, and Department of Microbiology and Plant Biology, University of Oklahoma, Norman, OK, USA

**Keywords:** Shock, Adaptation, Ethanol, Microevolution, Thermophile

## Abstract

**Introduction:**

The molecular links between shock-response and adaptation remain poorly understood, particularly for extremophiles. This has hindered rational engineering of solvent tolerance and correlated traits (e.g., productivity) in extremophiles. To untangle such molecular links, here we established a model that tracked the microevolution from shock to adaptation in thermophilic bacteria.

**Method:**

Temporal dynamics of genomes and transcriptomes was tracked for *Thermoanaerobacter* sp. X514 which under increasing exogenous ethanol evolved from ethanol-sensitive wild-type (Strain X) to tolerance of 2%- (X_I_) and eventually 6%-ethanol (X_II_). Based on the reconstructed transcriptional network underlying stress tolerance, genetic engineering was employed to improve ethanol tolerance and production in *Thermoanaerobacter.*

**Results:**

The spontaneous genome mutation rate (μ_g_) of *Thermoanaerobacter* sp. X514, calculated at 0.045, suggested a higher mutation rate in thermophile than previously thought. Transcriptomic comparison revealed that shock-response and adaptation were distinct in nature, whereas the transcriptomes of X_II_ resembled those of the extendedly shocked X. To respond to ethanol shock, X employed fructose-specific phosphotransferase system (PTS), Arginine Deiminase (ADI) pathway, alcohol dehydrogenase (Adh) and a distinct mechanism of V-type ATPase. As an adaptation to exogenous ethanol, X_I_ mobilized resistance-nodulation-cell division (RND) efflux system and Adh, whereas X_II,_ which produced higher ethanol than X_I_, employed ECF-type ϭ^24^, an alcohol catabolism operon and phase-specific heat-shock proteins (Hsps), modulated hexose/pentose-transport operon structure and reinforced membrane rigidity. Exploiting these findings, we further showed that ethanol productivity and tolerance can be improved simultaneously by overexpressing *adh* or ϭ^24^ in X.

**Conclusion:**

Our work revealed thermophilic-bacteria specific features of adaptive evolution and demonstrated a rational strategy to engineer co-evolving industrial traits. As improvements of shock-response, stress tolerance and productivity have been crucial aims in industrial applications employing thermophiles, our findings should be valuable not just to the production of ethanol but also to a wide variety of biofuels and biochemicals.

## Introduction

Adaptive evolution is a universal theme of life on our planet [1-3]. It is also a widely practiced strategy for selecting and engineering economically valuable traits [4-6]. Adaptive evolution typically starts from an environmental change and results in genetically inheritable adaptation [3,7]. The initial cellular response, which usually includes a transient reprogramming of cellular activities, is termed “shock”, while the subsequent cellular state that involves inheritable traits resulted from long-term exposure and selection (i.e., after generations) is termed “adaptation”. Numerous studies have investigated the cellular programs underlying shock (e.g., *Escherichia coli*[5,8,9]; *Saccharomyces cerevisiae*[10,11] and *Clostridium acetobutylicum*[12,13]) or adaptation [6,11,14-21], but few have attempted to test their links by tracking the temporal development from shock-response to the eventual adaptation [10,22,23]. The vast numbers of genetic variables (e.g., strains used) and environmental variables (e.g., culture conditions) across the studies hampered meaningful comparisons between shock- and adaptation-responses. Thus the molecular links between shock and adaptation are not yet well established, and the temporal characteristics and genetic mechanisms defining the shock-to-adaptation process remain elusive [10,11]. Moreover, whether and how the process was shaped by ecological parameters such as temperature is largely unknown.

Although most contemporary life forms are found at a narrow range of 24-40°C [24], the thermophiles thrive under optimal temperature of >50°C [24]. These organisms play a profound role in evolution and ecology of our biosphere (primordial life on earth is believed by many to be thermophilic [2]). They have also found wide applications in biotechnology. For example, thermophilic gram-positive anaerobes (TGPAs) such as certain *Thermoanaerobacter* and *Clostridium* species are of interest in producing solvents (e.g., ethanol, butanol and isopropanol) from lignocelluloses under a Consolidated Bioprocessing (CBP) scheme [25,26], due to their thermophilic nature (60-65°C), wide spectrum of carbon-sources [27,28] and co-utilization of pentose and hexose [25]. However, as is the case in many mesophiles, TGPAs are generally sensitive to excessive concentrations of their own solvent products [27], which reduce cell vitality, impair membrane integrity, inhibit enzymes and/or perturb intracellular pH balance [29,30]. Cellular tolerance to solvents can be derived by adaptive evolution of the wild-type strains via exposure to exogenous solvents for months or even years [30,31]. However, solvent-tolerant strains derived via such a strategy usually exhibit lower productivity of the solvent (although this reduction in yield was not proportional; [4,31]); this negative correlation between tolerance and productivity represents a major hurdle in strain development. In fact, although for many organisms genetic engineering of either solvent tolerance (e.g., [4,6]) or solvent productivity (e.g., [25,27,32]) has been accomplished, simultaneous improvement of solvent tolerance and productivity has not been demonstrated in thermophiles [4,33]. Devising a rational strategy to counter this hurdle might require a mechanistic understanding of the co-evolution of such linked traits.

Here we developed a model of adaptive evolution for thermophiles by evolving, under increasing concentrations of exogenous ethanol, a *Thermoanaerobacter* sp. X514 (NCBI Taxonomy ID: 399726) lineage from the ethanol-sensitive wild-type to tolerance of 2% ethanol, and eventually to 6%-ethanol tolerance. The genomes, transcriptomes and gene networks were traced and compared on a temporal scale, which unveiled the molecular links between shock and adaptation and revealed unusual features of “thermophilic” adaptive evolution. Furthermore, these findings enabled us to demonstrate that for wild-type TGPA strains, the two linked and co-evolving traits of ethanol productivity and ethanol tolerance could be simultaneously improved by genetic approaches (overexpressing an iron-containing Adh enzyme (Teth5140145-0146) or an ECF subfamily RNA polymerase sigma-24 factor regulator (ϭ^24^, Teth5141847-1848)).

## Results

### An experimental model of adaptive evolution from shock to adaptation in thermophilic bacteria

**Figure 1:**
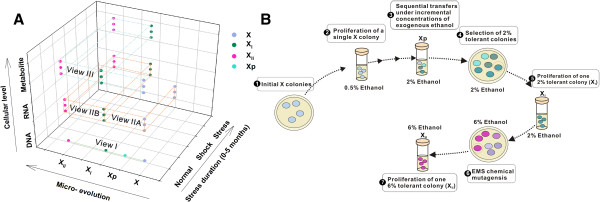
**Experimental strategy tracking the genotypes and phenotypes along the microevolution from ethanol shock to tolerance. (A)** Overview of experimental design. The three biological replicates for each sampled condition were indicated as dots. **(B)** Experiments to derive Xp (X_I_) and X_II_ that tolerated 2% and 6% (v/v) ethanol respectively. EMS: ethylmethane sulfonate. Xp: the mixed culture which grew under 2% ethanol and from which X_I_ was isolated.

**Figure 2:**
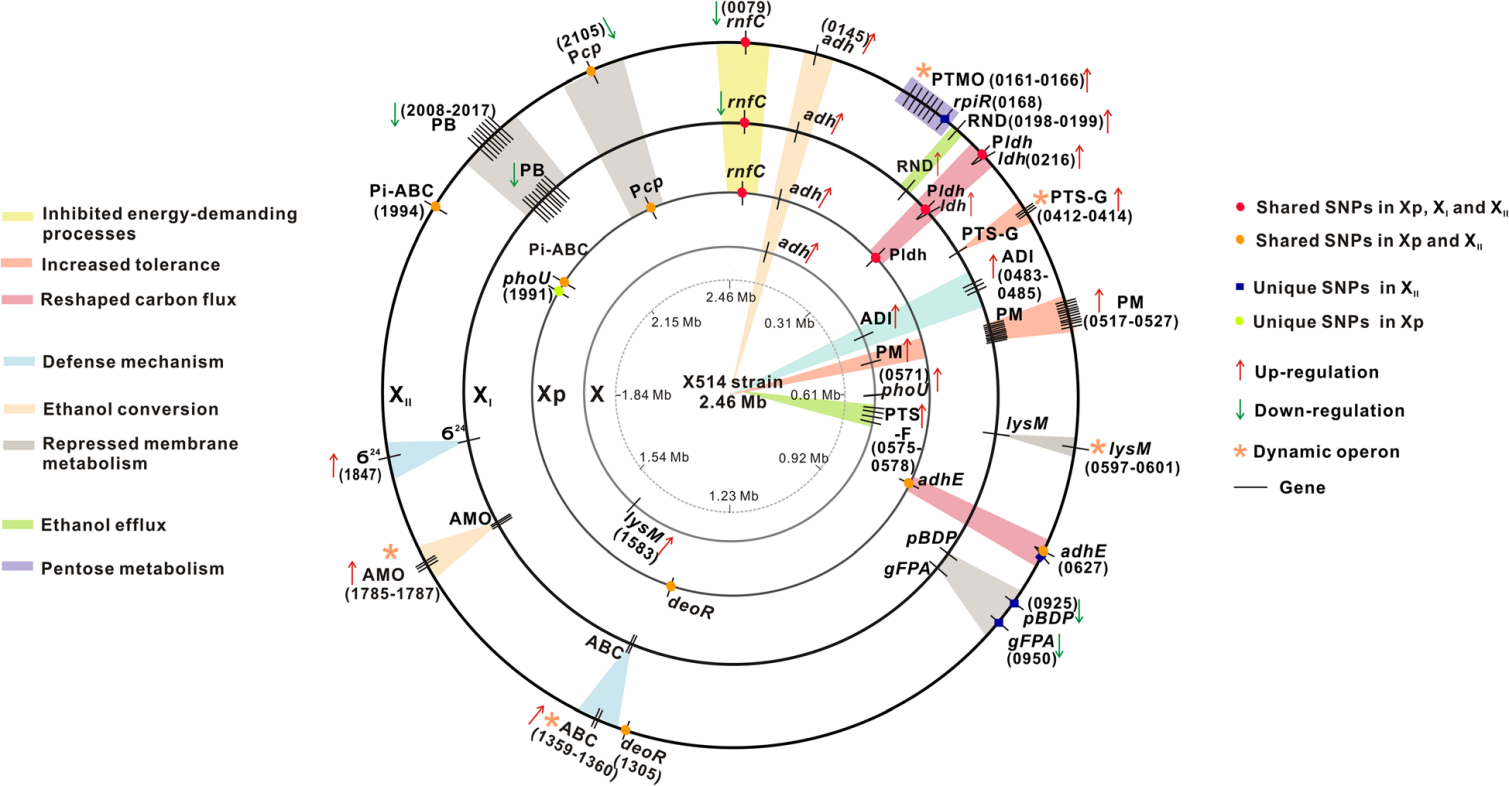
**Genomic and transcriptomic events defining each developmental phases over the complete time-course.** Key mutated genes, gene with significantly changed expression levels and dynamic operons were illustrated. P*ldh*: the promoter of lactate dehydrogenase; PTMO: pentose transport and metabolism operon; PM: purine metabolic genes; PTS-G/F: glucose/fructose specific PTS system; *pBDP*: peptidoglycan binding domain-containing protein; *gFPA*: glucosamine--fructose-6-phosphate aminotransferase; AMO: alcohol metabolism operon; Pi-ABC: phosphate ABC transporter; PB: peptidoglycan biosynthesis; P*cp*: the promoter of Serine-type D-Ala-D-Alacarboxypeptidase. IDs of the corresponding genes in X514 were shown.

**Figure 3:**
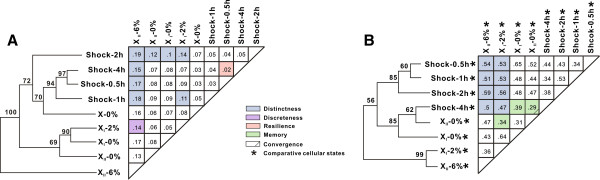
**Links among the cellular states of shock and tolerance as defined by transcriptome. (A)** Links among the nine cellular states. **(B)** Links among the eight relative cellular states. The number in each square represented the distance between each pair-wise comparison as calculated based on Pearson Correlation. Hierarchical clustering of the nine cellular states and the eight relative cellular states were shown respectively. Numbers on the branches represent bootstrap values as percentages of 1000 resampling efforts of the dataset. The clustering analysis was performed by TM4 software [57].

*Thermoanaerobacter* sp. X514 is a TGPA we previously used for dissecting the mechanism of pentose-hexose co-utilization [25] and ethanol production [34]. We first designed a trackable experimental model of adaptive evolution for thermophiles (Figure 1A and 1B) by exploiting the observed ethanol-sensitivity of wild-type X514 (designated “X”; Additional file 1A) to the mutant strain X_I_ (“low ethanol tolerance”, i.e., tolerating 2% ethanol; Additional file 2A; Methods) that was one isolate of Xp (a mixed culture of mutants that were developed from X via sequential transfer) and eventually to another mutant strain X_II_ (“high-ethanol tolerance”, i.e., tolerating 6% ethanol; Additional file 2A; Methods). The three developmental phases of ethanol tolerances represented by X, X_I_ (and Xp) and X_II_ were interrogated under three “Views” (Figure 1A): (i) Phenotypic adaptation (in X, X_I_ and X_II_) (View I; Additional file 2). (ii) Transcriptomic responses that respectively defined four time-points under ethanol shock and the two phases of ethanol adaptation (View II; Figures 2 and Figure 3). Under the particular culture medium, the ethanol concentration that caused stress but not significant cell death in X was 0.15% (v/v), which was selected for the ethanol shock assay (Additional file 1A). Thus, subsequent shock responses were examined by comparing X-0.15% (X cells cultured at defined medium with 0.15% exogenous ethanol; View IIA) to X-0% (X cells cultured without exogenous ethanol); moreover, dynamics of shock responses was tested by sampling X transcriptomes at four time points (0.5 h, 1 h, 2 h and 4 h) upon ethanol exposure. On the other hand, mechanism of ethanol tolerance was revealed by the transcriptomes of X_I_ (X_I_-0% and X_I_-2%; X_I_ cells cultured under 0% and 2% ethanol respectively) and those of X_II_ (X_II_-0% and X_II_-6%; X_II_ cells cultured under 0% and 6% ethanol respectively; View IIB). In addition to microarray-based expression profiling, RNA-Seq was employed so as to detect all structural and sequence changes of transcripts during the microevolution. (iii) Genome sequence mutations (for X, Xp, X_I_ and X_II_) (View III; Figure 2). To precisely identify every genotypic change along the microevolution, we resequenced the X, X_I_ and X_II_ genomes and the metagenome of Xp, using the previously finished wild-type X514 genome [34] as reference.

Higher concentrations of exogenous ethanol slowed down growth in all strains (Additional file 2A), with reduction in cell length correlated with improvements in tolerance (Additional file 3). Glycolysis in the tolerant mutants was as robust as that in the wild-type yet the carbohydrate metabolism was altered, as in the absence of exogenous ethanol, X_I_ and X_II_ consumed more glucose (100:120:115 for X: X_I_: X_II_) yet produced less ethanol (100:45:70 for X: X_I_: X_II_) than X (Additional file 2B).

In Xp, X_I_ and X_II_ genomes (in reference to X; Methods), 76 (74 single-nucleotide polymorphisms (SNPs) and two single-base indels), 20 (19 SNPs and one single-base indel) and 45 (43 SNPs and two single-base indels) mutations were respectively found (Figure 2; Additional file 4). Along the microevolution, the ratio between non-coding sequence mutations and those in coding sequence stayed largely unchanged (from 17.6% in X_I_ to 15.4% in X_II_); moreover the functional profile of non-synonymous mutations shifted from regulatory functions to structural proteins (e.g., membrane and sugar phosphate metabolisms; Additional file 4). On the other hand, in X, X_I_ and X_II_ transcriptomes, expression-altered genes were mostly concentrated in amino acid metabolism, cell motility, coenzyme metabolism, carbohydrate metabolism and energy production (Additional file 5). Along the microevolution, the functional profile of expression-altered genes shifted from one mainly featuring amino acid metabolism and cell motility to one primarily of energy production, suggesting ethanol adaptation but not shock compromised energy generation. Together, the microevolution featured decreasing numbers of expression-altered genes (314 genes under X_I_-0% vs X-0% and 189 genes under X_II_-0% vs X-0% were significantly changed) yet increasing numbers of DNA-sequence changes (20 mutations in X_I_ and 45 in X_II_), suggesting a temporal shift from changes in gene expression to changes in genome sequence (Figure 2, Additional file 5 and Additional file 4). Interestingly, expression alteration and DNA-sequence changes exhibited complementary functional landscapes. In X_I_, the former were mainly found in amino acid, coenzyme and carbohydrate metabolisms, yet the latter mainly involved regulatory functions (transcription factors (TFs) and *cis*-regulatory elements) (Figure 2 and Additional file 5). In X_II_, expression alteration mainly involved carbohydrate metabolism, energy production and amino acid metabolism, while DNA-sequence changes mainly involved membrane and sugar phosphate metabolism genes and TFs (Figure 2 and Additional file 5).

Comparison between X_I_ and X genomes revealed a spontaneous mutation rate per genome per generation (μ_g_) of 0.045 given 440 generations. For another thermophile *Thermusthermophilus*, under its optimal growth condition, a μ_g_ of 0.00093 was proposed based on mutation reporter gene *pyrEF*[35], which was nearly 10-fold lower than the mesophile *E.coli* (0.0048); the much lower μ_g_ in thermophiles (mean μ_g_ 0.00079, range 1.4-fold) than mesophiles (mean μ_g_ 0.0040, range 2.9-fold) was interpreted as due to the rapid accumulation of deleterious mutations at a temperature only 5-10^o^C higher [35]. However, under ethanol stress, our experimentally estimated μ_g_ of 0.045 for X514 appeared to be two orders of magnitude higher than that of optimal-growth *Thermusthermophilus* and actually slightly higher than that of *E.coli* under isobutanol stress (0.026) [5], contradicting with the current notion [35].

### Relationship among transcriptomic programs that respectively underlie shock, low-tolerance and high-tolerance

Relationship among the four shock-stages (X-0.15% at 0.5h, 1h, 2h and 4h upon exogenous-ethanol exposure) and the four adaptation states (X_I_-0%, X_I_-2%, X_II_-0% and X_II_-6%) were unveiled by pair-wisely comparing the nine transcriptomic states (including X-0%; each state in biological triplicates; Figure 3A; Methods). Three observations were apparent. (i) Shock-response and adaptation were distinct in nature (i.e., “Distinctness”), as mutant states (X_I_ and X_II_) and X states formed two separate clusters, with large pairwise distances between them (mostly over 0.1). (ii) Adaptation was phased (i.e., “Discreteness”), as X_II_-6% formed an independent clad with a large pairwise distance (0.14) between X_II_-6% and X_I_-2%. (iii) For both shock-response and adaptation, their temporal development exhibited a certain degree of “Resilience”. From the onset of environmental change, the transcriptomes were first altered to a distinct state but then returned to one that was closer to the original state (Figure 3A, Additional file 1B and Additional file 5): for example, in shock, X-4 h was closer than both X-1 h and X-2 h to X-0.5 h; similarly, in adaptation, X_II_-0% was closer to X-0% than X_I_-0%.

Furthermore, to distinguish between environmental and genetic effects, the relative transcriptomic changes that included the six “normalized” transcriptomes due to environmental perturbation (X-0.15% vs X-0% at each of 0.5 h, 1 h, 2 h and 4 h; X_I_ -2% vs X_I_-0%; X_II_-6% vs X_II_-0%) and the other two caused by genetic changes (X_I_ -0% vs X-0%; X_II_-0% vs X-0%) were pair-wisely compared (Figure 3B; Methods). Two findings emerged. (i) Changes of the cellular states corresponding to the evolving tolerance exhibited a degree of “Memory” left by *a priori* exposure to the stimuli, as demonstrated by *a*) the similarity between X_I_-0% (vs X-0%) and X-0.15%-4h (vs X-0%-4h), and *b*) the similarity between X_II_-0% (vs X-0%) and X_I_-2% (vs X_I_-0%) (Figure 3B). (ii) The shock and adaptation programs appeared progressing towards a shared destiny of cellular state, as the shortest pair-wise distance (at 0.296) among the 28 such distances was actually found between the high-tolerance stage (X_II_-0% (vs X-0%)) and the wild-type under the most extended shock (X-0.15%-4h (vs X-0%-4 h)) (Figure 3B). This suggested “Convergence”, where the high-tolerance cells, in the absence of stimuli, retained certain transcriptomic features of those under extended shock.

### A dynamic yet coordinated response to ethanol shock (View IIA)

#### Ethanol-shock networks

How gene networks of thermophiles respond to environmental stimuli has been poorly understood [36]. For X, two genome-wide gene co-expression networks respectively characterizing ethanol-shock cells (ES^+^) and control cells (ES^-^) were constructed via co-expression analysis and then compared (Methods; Additional file 6). ES^+^ (216 nodes; 45 of them were hypothetical proteins representing new components of ethanol-shock response; Part I of Additional file 7; Additional file 6) was 23.7% smaller than ES^-^ (283 nodes), with eleven modules each of at least five nodes found in each network (module sizes vary substantially both within and between networks, ranging from 5 to 93 nodes). There were 30 ES^+^–specific (e.g., small multi-drug export, channel protein, cell wall hydrolase and ethanolamine utilization protein EutN) and 97 ES^-^–specific nodes (electron complex and ribosomal proteins), suggesting repression of energy metabolism and protein translation and activation of detoxification under shock.

**Figure 4:**
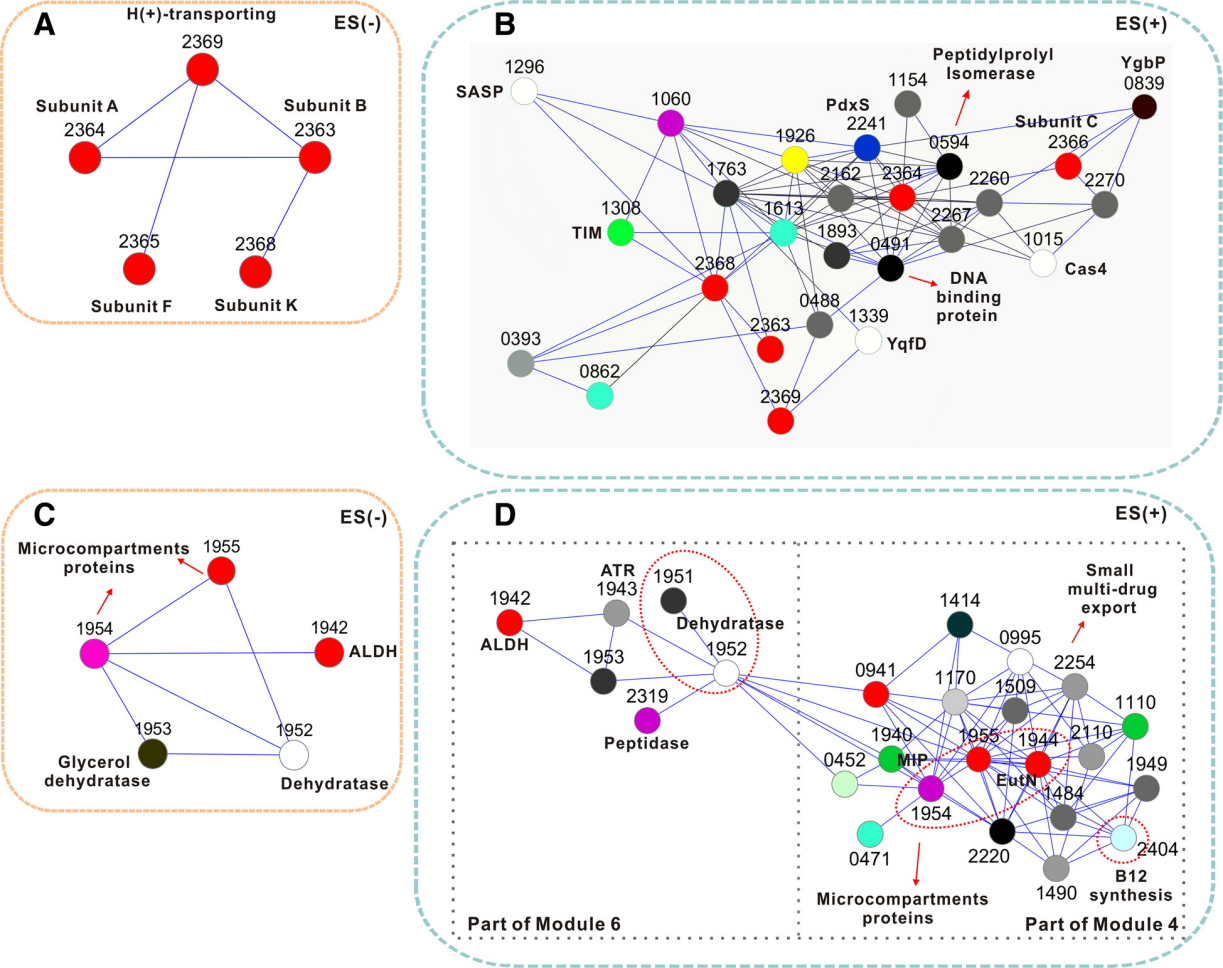
**Gene co-expression network of the wild-type strain under ethanol shock.(A)** The sub-module of V-type ATPase under control (ES^-^). **(B)** The sub-module of V-type ATPase under ethanol shock (ES^+^). Only the first neighbors (genes directly connected to the V-type ATPase genes) were shown. **(C)** The sub-module of genes involved in defense mechanism under control (ES^-^). **(D)** The sub-module of genes involved in defense mechanism under ethanol shock (ES^+^). Color code was as in Additional file 6. Blue lines indicated positive correlation.

Moreover, for the 186 nodes shared between ES^+^ and ES^-^, inter-node relationships were distinct. Among the top twenty such nodes with the most connections in ES^+^, several were known to play pivotal roles in ethanol shock. The first group was V-type ATPase, which maintains intracellular pH homeostasis in *S. cerevisiae* upon ethanol shock [11]. In ES^-^, these genes (Teth5142363-2365 and Teth5142368-2369) constituted a module (Module 10) free of inter-module links (Figure 4A and Additional file 6; also observed in our recently reported *Thermoanaerobacter* glycobiome network [25]). However, in ES^**+**^, these genes became connected with other genes; not only were they found in the largest module (Module 1; with 59 nodes) (Additional file 6), but also directly linked to genes protecting cells from organic solvent damages or related to membrane structure (Figure 4B; Part I of Additional file 7).

The second group consisted of genes that metabolize or exclude toxic compounds. In ES^-^, Module 11 (5 genes) was free of connections with other modules, indicating these five genes (aldehyde dehydrogenase (Teth5141942, *aldh*), microcompartment proteins (Teth5141954-1955) and dehydratases (Teth5141952-1953) (Figure 4C and Additional file 6) formed a single functional unit to metabolize toxic intermediates [37]. Interestingly, in ES^+^, the unit expanded to include 25 genes organized into two modules (Module 4 and 6) (Figure 4D and Additional file 6): Aldh converts acetaldehyde (the toxic product of ethanol oxidation) to acetate; Teth5142404 participates in vitamin B_12_ synthesis; Teth5141943 (ATP-cobalaminadenosyltransferase; converting B_12_ to coenzyme B_12_) provides the essential coenzyme to B_12_-dependent microcompartment protein (Teth5141944); the latter and additional such proteins (Teth5141954-1955) remove extra aldehyde through major intrinsic protein (MIP) channel (Teth5141940); multi-drug exporter (Teth5142254) excludes toxic chemicals; peptidase S51 (Teth5142319) degrades misfolded proteins and prevents their accumulation. Several (Teth5141940, Teth5141943-1944 and Teth5142254) of these genes were absent in ES^-^.

#### Dynamics of ethanol-shock networks

Transcriptomes respectively sampled at 0.5 h, 1 h, 2 h and 4 h upon ethanol-shock revealed a total of 520 genes differentially expressed (X-0.15% vs X-0%) in at least one time-point (|log_2_*R*| ≥1; Additional file 8). Their expression patterns formed ten temporal clusters (Additional file 9) that include both stimulatory and inhibitory responses. An iron-containing *adh* (Teth5140145; one of the nine *adh* genes in X514 genome) and ADI Pathway (Cluster 9) [25,38] were the earliest tide, peaking at 0.5h and then quickly subduing. The second (Cluster 7) surged at 1h, among which was the small acid-soluble spore protein gene (Teth5141739) that protects DNA backbone from chemical and enzymatic cleavage. The third (Cluster 3) was induced at 2 h and included *lysM* (Teth5141583) which encodes a general peptidoglycan binding function and resists ethanol damage [39]. Cluster 1, consisting of purine metabolism, fatty acid metabolism and fructose PTS genes, maintained upregulation within 2 h. On the other hand, several clusters were downregulated. Amino acid metabolism genes (valine, arginine and tyrosine; Cluster 2) represented the earliest inhibited genes (from 0.5 h to 4 h), followed by histidine metabolism and carbohydrate transport regulators (Cluster 5; inhibited from 1 h to 4 h). Subsequently, dipeptide transport, ion transport, carbohydrate transport and flagella synthesis fell during 2 h to 4 h (Clusters 4 and 6; although Cluster 4 later restored to the control level), followed by ribosomal proteins, DNA replication and carbohydrate metabolism genes (Clusters 8 and 10) that were repressed at 4 h. Prolonged ethanol exposure extending from 0.5 h to 4 h resulted in dramatic increase of downregulated genes (from 37 to 350 genes; 14.1% of genome) and decrease of upregulated genes (from 40 to 4 genes) (Additional file 1B).

Therefore, upon ethanol shock, X mobilized a highly dynamic yet coordinated program organized in ten temporal clusters and involved dozens of upregulated genes (Additional file 9), however it was not clear whether any among them contributed to ethanol tolerance. Thus we next examined the 2%- and 6%-ethanol-tolerant mutants.

### Adaptation strategy of the low-tolerance mutant

#### Genome mutations in X_I_ and Xp

Mutations can be beneficial, neutral or deleterious [1]. In X_I_, 20 SNPs were identified that included 17 SNPs (6 nonsense and 11 missense) in coding and 3 in non-coding sequences (Additional file 4). Among them, six SNPs in coding regions were synonymous and thus likely neutral. To distinguish beneficial SNPs in X_I_, we sequenced the metagenome of Xp to a depth equivalent to 140 sequence coverage of X514 genome, the pool of mutants that tolerated 2% ethanol, reasoning that non-synonymous mutations with higher mutation frequency (percentage of mutated reads to all reads at a single-base locus in the X514 mutant community) were more beneficial (Additional file 10B).

Three such X_I_-mutations were present in Xp (Part II of Additional file 7) with high frequency (>80%), implicating energy conversion (COG C, electron transport complex I (Teth5140079) and lactate dehydrogenase (Ldh, Teth5140216)) and ion transport process (COG P, TrkH family potassium uptake protein (Teth5140140)) (Figure 2, Additional file 10C, Additional file 11 and Additional file 12; Part II of Additional file 7). Among them, one insertion mutation was found between the -10 box and the -35 box of the predicted promoter of an *ldh* (Teth5140216) that catalyzes lactate formation (Figure 2 and Additional file 11). The distance (but not the sequence) between these two conserved elements is crucial for regulating gene expression [40]; thus this regulatory adaptation likely led to *ldh* upregulation which was consistent with the increased *ldh* expression level (Figure 2) and then elevated lactate production in X_I_ (Additional file 2B).

#### A priori ethanol stress reshaped gene network of the cell

Comparison between X_I_-0% and X-0% revealed that *a priori* long-term ethanol exposure reshaped metabolisms, even in the absence of exogenous ethanol (Additional file 13A and Additional file 14B). *First*, central carbon metabolisms were transcriptionally altered. Corresponding to the one insertion mutation in promoter, one *ldh* (Teth5140216; the only *ldh* in X514 genome) was induced, whereas solvent formation genes were inhibited that included butyrate kinase (Teth5140936), phosphate butyryltransferase (Teth5140937), and iron-containing *adh* and its associated NADH oxidase (Teth5140145-0146) (Additional file 15A). In addition, several glycolysis and pentose phosphate pathway (PPP) genes (Teth5140161, Teth5140163-0165, Teth5140575-0576 and Teth5141896; Additional file 15A) were induced. Thus glycolysis in tolerant mutants was robust with carbon flux shifting from ethanol- to lactate-deriving, explaining the increased glucose consumption and elevated lactate production (Additional file 2B). *Second*, additional solvent formation genes were suppressed in mutants. For example, B_12_ dependent *eut*s (Teth5141943-1946), whose expression level positively correlates with ethanol production in X [25], were downregulated in X_I_, likely contributing to the decrease in ethanol titer (Additional file 2B). *Third*, coenzyme B metabolism was either up- (B_1_, B_2_ and B_5_) or down-regulated (B_12_; Additional file 15A). B_12_ biosynthesis (Teth5140298-0320) and related cobalt transporters (Teth5141931-1934 and Teth5140297-0326), which provide coenzyme B_12_ to ethanol formation genes [25], were all downregulated, leading to the lower ethanol production (Part III of Additional file 7). *Fourth*, genes in several stress-response pathways [10,41] were upregulated in X_I_ (Part III of Additional file 7**)**. Among them was up-regulation of NAD/NADP octopine dehydrogenase (Teth5142108; Additional file 15A). This multifunctional enzyme catalyzed the reversible reductive condensation of arginine and pyruvic acid to D-octopine [42]. The arginine can protect cells against ethanol damage [25]. On the other hand, the octopine dehydrogenase activity was significantly correlated with the ability to buffer the acidic end products of anaerobic metabolism in the marine invertebrate cephalopods [42]. As more lactate acid was produced in X_I_ (Additional file 2B), higher activity of this gene probably led to acid-damage resistance. *Finally*, nitrogen metabolism and cell wall/membrane metabolism were perturbed in X_I_ (Part III of Additional file 7).

#### Long-term ethanol stress changed the cellular stress-response program

**Figure 5:**
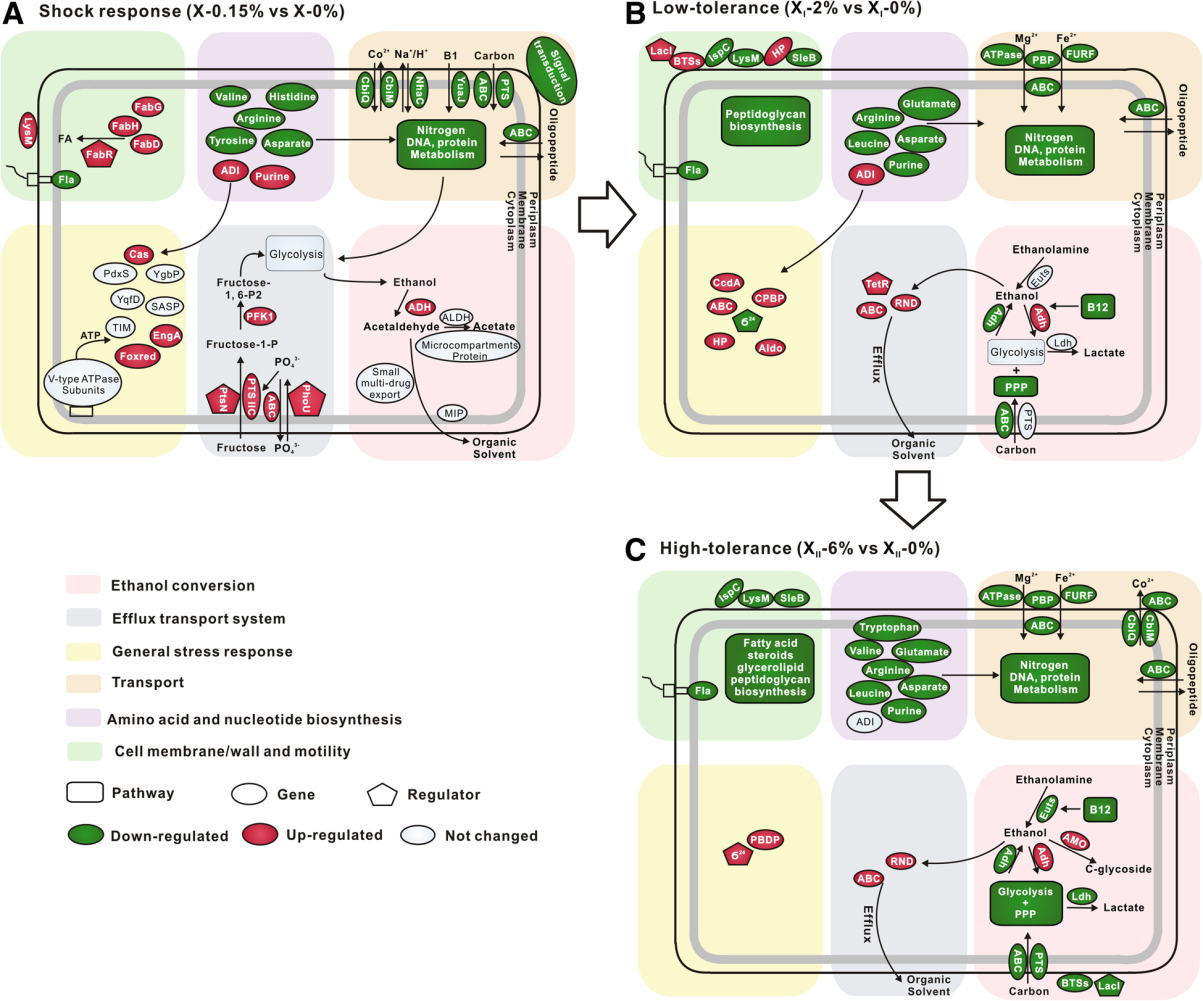
**Molecular events underpinning development of ethanol tolerance in thermopiles.** The transition from the shock response in X **(A)** to the low-tolerance in X_I_**(B)** and eventually to the high-tolerance in X_II_**(C)** was shown. Key transport, metabolic and regulatory genes and pathways were illustrated. Fla: flagellar biosynthesis; BTSs: binding-protein-dependent transport systems; Aldo: aldo/ketoreductase; PBD: peptidoglycan binding domain-containing protein; CcdA: cytochrome c biogenesis protein; TSPP: transport system permeaseprotein.PBP: periplasmic binding protein; IspC: cell wall hydrolase/autolysin; CPBP: capsule polysaccharide biosynthesis protein; PBDP: peptidoglycan binding domain-containing protein; FURF: ferric uptake regulator family protein; HP: hypothetical protein.

Comparison between X_I_-2% and X_I_-0% revealed how *long-term* stress altered the stress-response program (View IIB; Figure 5B and Additional file 14A). Overall, X_I_-2% featured an inhibited metabolism, including carbon metabolism (PPP pathways), energy conversion (e.g., acetate kinase (*ak*), *adh*, and 3-isopropylmalate dehydrogenase (*ipmdh*)), cell membrane metabolism, DNA metabolism, coenzyme biosynthesis (B_1_, B_2_, and B_12_), amino acid synthesis and cell motility (Additional file 16A). However, several genes were upregulated. (i) Extracellular solute-binding protein and binding-protein-dependent transport systems inner membrane component in COG G (Teth5142194-2202, Teth5140534 and Teth5141044) were upregulated (Figure 5B and Additional file 14A), however the associated carbohydrate transport systems were not induced, suggesting changed cell surface interactions but not increased carbohydrate transports. (ii) Efflux systems were elevated that likely removed intracellular ethanol, including tetracycline repressor (TetR) family transcription factor [43], RND family efflux transporter [44] and capsule polysaccharide biosynthesis [45] (Additional file 16A). (iii) An iron-containing *adh* (Teth5140145) and the ADI pathway (Teth5140483-0485) were activated.

Therefore in both X and X_I_, ethanol inhibited carbohydrate, amino acid and flagellar synthesis. The defense mechanisms shared between shock and low-tolerance included the *adh* operon that controls intracellular ethanol [25] and ADI pathway that maintains pH balance [25,38]. On the other hand, X appeared to employ fructose-specific PTS systems for extruding ethanol [46], whereas X_I_ mobilized RND and TetR presumably to activate efflux pumps for ethanol [43] and extracellular solute-binding protein to tune cell-surface interactions (Figure 2, Figure 5A-B and Additional file 14A).

### Adaptation strategy of the high-tolerance mutant

#### Genome mutations in X_II_

The three apparently beneficial mutations of X_I_, found at electron transport complex I, *ldh* promoter and TrkH family potassium uptake protein respectively, were also found in X_II_ (Figure 2 and Additional file 10D), consistent with X_II_ being derived from X_I_. Moreover, 12.1% of non-synonymous mutations in X_II_ were found in Xp but not in X_I_, indicating these mutations might further enhance ethanol tolerance (Figure 2 and Additional file 10D; Part IV of Additional file 7). In both Xp and X_II_, Leu^**598**^?→?Gln was found in the Fe-ADH domain of AdhE (bifunctional aldehyde/alcohol dehydrogenase; Additional file 17B). Furthermore, in X_II_, one more SNP (His^**748**^?→?Arg) was identified in this domain (near the active-site iron and the cofactor-binding sites), likely altering the interaction between the ADH-domain and NADP (Additional file 17B; Methods) [4]. In addition, in X_II_, analogous to the mutated RodA (Teth5142127) in Xp, another rod shape-determining protein, MreC, was mutated (Val^**132**^?→?Gly, Teth5142133). MreC plays a role in determining cell shape (e.g., the rod-shape in *E. coli*[47]), likely underlying the shortened length of X_II_ cells (Additional file 3).

X_II_ harbored additional SNPs that were absent in both Xp and X_I_. They were mostly in two categories: ribose metabolism (e.g., Thr^**94**^?→?Ala in RpiR) and cell membrane metabolism (Figure 2 and Additional file 10C; Part IV of Additional file 7). Thus the altered ribose and membrane metabolism in X_II_ might contribute to the enhanced tolerance.

#### Features of the cellular state of X_II_

Transcriptomic comparison of X_II_-0% versus X-0% revealed characteristics of X_II_ state, including altered carbon-metabolisms (inhibited *adh* cluster (Teth5140145-0146) in glycolysis and induced oxaloacetate decarboxylase (*oad*, Teth5141582; converting oxaloacetate into pyruvate)) in pyruvate metabolism, induced stress response, repressed nitrogen metabolism and inhibited cell wall/membrane metabolism (Additional file 13B and Additional file 18; Part IV of Additional file 7). Notably, *fabR* (Teth5141728), a global regulator of membrane lipid biosynthesis in many Gram-positive bacteria [48], was up-regulated in X_II_-0%, together with *teth5141723-1727*. *FabG* (Teth5141723) and *fabD* (Teth5141724) were involved in long chain fatty acid biosynthesis, whereas *fabH* (Teth5141726) was the determining factor in branched-chain fatty acid biosynthesis [49]. Therefore, X_II_ reinforced membrane rigidity against ethanol damage [30]. Such expression patterns were also observed under ethanol shock in X at 1h and 2h, revealing a link between shock response and adaptation that likely underlay the observed “Memory” effect in this adaptive evolution (Figure 3B). In addition, the induction of heat shock proteins (Hsps, which were a universal response to ethanol stress in mesophiles) was not observed under either the shock to X (X-0.15% vs X-0%) or the stress to the mutants (X_I_-2% vs X_I_-0% and X_II_-6% vs X_II_-0%) (Part V of Additional file 7), suggesting one TGPA-specific feature of adaptive evolution.

#### Adaptation from low-tolerance to high-tolerance

Comparison of X_II_-0% and X_I_-0% transcriptomes explained the increased tolerance yet higher ethanol productivity in X_II_*.* Higher ethanol-tolerance generally correlated with lower ethanol-productivity, as X_I_ and X_II_ produced less ethanol than X (Additional file 2B), which was due to rewired glycolysis (X_I_ and X_II_), suppressed solvent formation genes (X_I_ and X_II_) and inhibited co-enzyme B biosynthesis (X_I_). Surprisingly, X_II_ both tolerated and produced higher ethanol than X_I_ (Additional file 2B; *p* = 0.01), suggesting positive co-evolution of the two traits under certain circumstances (Additional file 8 and Additional file 14B).

In X_II_-0% (vs X_I_-0%), 21.6% (535) of the genes were upregulated, while 1.3% (33 genes) downregulated. Upregulation was mostly in three categories. First, solvent formation genes (6.54%) were induced, which included *eut*s (Teth5141940-1946), *de novo* B_12_ biosynthesis (Teth5140299-0320) and butyrate biosynthesis (Teth5140936-0944). These likely underpinned the higher-than-X_I_ ethanol-production of X_II_.

Second, among the carbon/ion transporters repressed in X_I_ and X_II_ (X-0% as baseline), 51 genes (10% of all upregulated genes) were expressed higher in X_II_ than X_I_ (e.g., Teth5140323-0326 for cobalt transport in B_12_ biosynthesis), indicating a lesser degree of inhibition in X_II_. Notably, dynamically regulated operon structures were observed in these upregulated carbon transporters between X_I_ and X_II_, including pentose transport (Teth5140161-0166) and glucose transport (Teth5140412-0414) (Figure 2 and Additional file 19). In X_I_, genes involved in pentose transport were clustered in a single operon (Additional file 19A), as was the case for glucose transport genes (Additional file 19C). However, in X_II_, pentose transport genes were transcribed in three suboperons (Additional file 19B), whereas glucose transport genes formed two suboperons (Additional file 19D). Such dynamic operon structures might be a mechanism to precisely tune the ratios of enzyme-encoding transcripts as an adaptation strategy specific at the high-tolerance phase.

Third, general stress-response (5.2% of all upregulated genes; [36,41]) was specifically induced in X_II_-0% (vs X_I_-0%). It included ABC transporters, Hsps (e.g., DnaK, GrpE, and Hsp20), peptidases, CRISPRs-associated (Cas) immunity system, ADI pathway, and oxidoreductases. It also included several genes induced in X_I_-2% but not in X_I_ -0% (vs X-0%; i.e., the “Memory” of X_II_-0%), such as the TetR regulator in efflux systems and the aldo/ketoreductase in oxidoreduction (Additional file 12B). Interestingly, Teth5141359-1361, an ABC transporter operon in COG V (defense mechanism) was “dynamic”. In X_I_, Teth5141359-1361 constituted a single operon (Additional file 19E). However, in X_II_, they formed two suboperons, with the first (Teth5141359-1360, encoding hypothetical protein and ABC transporter) up-regulated in X_II_-0% (vs X_I_-0%) while the second (Teth5141361, a hypothetical protein) not induced (Additional file 19F), suggesting dynamic operon as a mechanism for gene-specific upregulation within an operon.

#### The distinct stress-response program of X_II_ and its link to X_I_

Comparison between X_II_-6% and X_II_-0% demonstrated how long-term stress shaped the stress-response program in the high-tolerance phase (Figure 5C). In X_II_-6%, despite the downregulation of 1583 genes, 16 were upregulated (Additional file 16B). (i) One extracytoplasmic function (ECF) subfamily ϭ^24^ locus (Teth5141847-1848; Figure 5C and Additional file 14A) was induced, which encodes RNA polymerase subunits that regulate intracellular responses to various extracellular stimuli [41]. In *Bacillus subtilis*, this ECF ϭ (SigM) was activated under ethanol shock and salt stress [50]. (ii) RND family efflux system operon (Teth5140198-0200) was upregulated, which extrudes ethanol [44]. (iii) The iron-containing *adh* (Teth5140145-0146) operon and the alcohol catabolism operon (Teth5141785-1787), which convert ethanol into other intermediate metabolites, was expressed higher in X_II_-6% than X_II_-0%. The Teth5141786 activated nucleotide sugar and then Teth5141787 transferred glycosyl from nucleotide sugar to alcohol, forming C-glycoside to reduce the intracellular alcohol concentration. Interestingly, in X_II_-6%, due to functional correlation, they constituted one operon, instead of two suboperons (Teth5141785-1786 and Teth5141787) in X_II_-0% (Additional file 20), suggesting dynamic operons can be condition-dependent. (iv) Peptidoglycan binding domain-containing protein (Teth5140954) in COG V (defense mechanism) was induced, which can protect cell wall from autolysis.

Comparison between X_II_-6% and X_I_-2% revealed 72 (3%) upregulated genes and 725 (29%) downregulated genes (Figure 2 and Additional file 14A; Part VI of Additional file 7), which are key to the 2%-to-6% tolerance improvement. The 72 upregulated genes include, (i) iron-containing *adh* (Teth5140145-0146) operon, which suggested positive correlation between ethanol conversion and tolerance; (ii) purine metabolism operons (Teth5140517-0525 and Teth5142354-2355), which was also upregulated in X under ethanol shock and thus was a shock-adaptation link (Additional file 14A); (iii) cell wall and membrane biosynthesis genes (Teth5141784-1789, Teth5140797-0799 and Teth5141976); (iv) *hsps*(including *dnaK*, *dnaJ*, *grpE* and *hrcA*; Teth5142078-2081), which were upregulated under 6%-ethanol stress (X_II_-0% vs X_I_-0%; X_II_-6% vs X_I_-2%) but not under shock or 2%-ethanol stress (X-0.15% vs X-0%; X_I_-2% vs X_I_-0%), suggesting their phase-specific functioning; (v) the ϭ^24^ (Teth5141847) which was also a unique feature of X_II_ upon ethanol stress; (vi) ribosome protein genes (Teth5140864-0881), which suggested a potential contribution of translation machineries in X_II_ tolerance; (vii) transporter genes including ABC transporter (Teth5140253-0254, COG P), Na^+^/H^+^ exchanger (Teth5141320, COG P) and cellobiose specific PTS IIA (Teth5140265), which were repressed by ethanol in both X_I_ and X_II_ (X as baseline) yet the inhibition was alleviated in X_II_-6% (vs X_I_-2%), potentially explaining the growth of X_II_ but not X_I_ under 6% ethanol.

Thus X_I_ and X_II_ mobilized linked yet distinct defense mechanisms (Figure 2 and Figure 5). The former included RND efflux system and *adh* operon, both controlling intracellular ethanol. For the latter, X_I_ mobilized TetR presumably to activate efflux pumps for ethanol [43] and ADI pathway to maintain pH balance [25], whereas X_II_ employed ϭ^24^, alcohol metabolism operon and peptidoglycan binding domain-containing protein (Teth5140954).

### Exploiting the molecular links between shock response and adaptation for simultaneous improvement of ethanol tolerance and titer

The higher ethanol titer and higher tolerance in X_II_ than X_I_ suggested ethanol production level was not necessarily negatively correlated with ethanol tolerance. The unraveled links and distinctions among the stress-response programs in X, X_I_ and X_II_ provided rational genetic strategies to engineer the two co-evolving traits.

**Figure 6:**
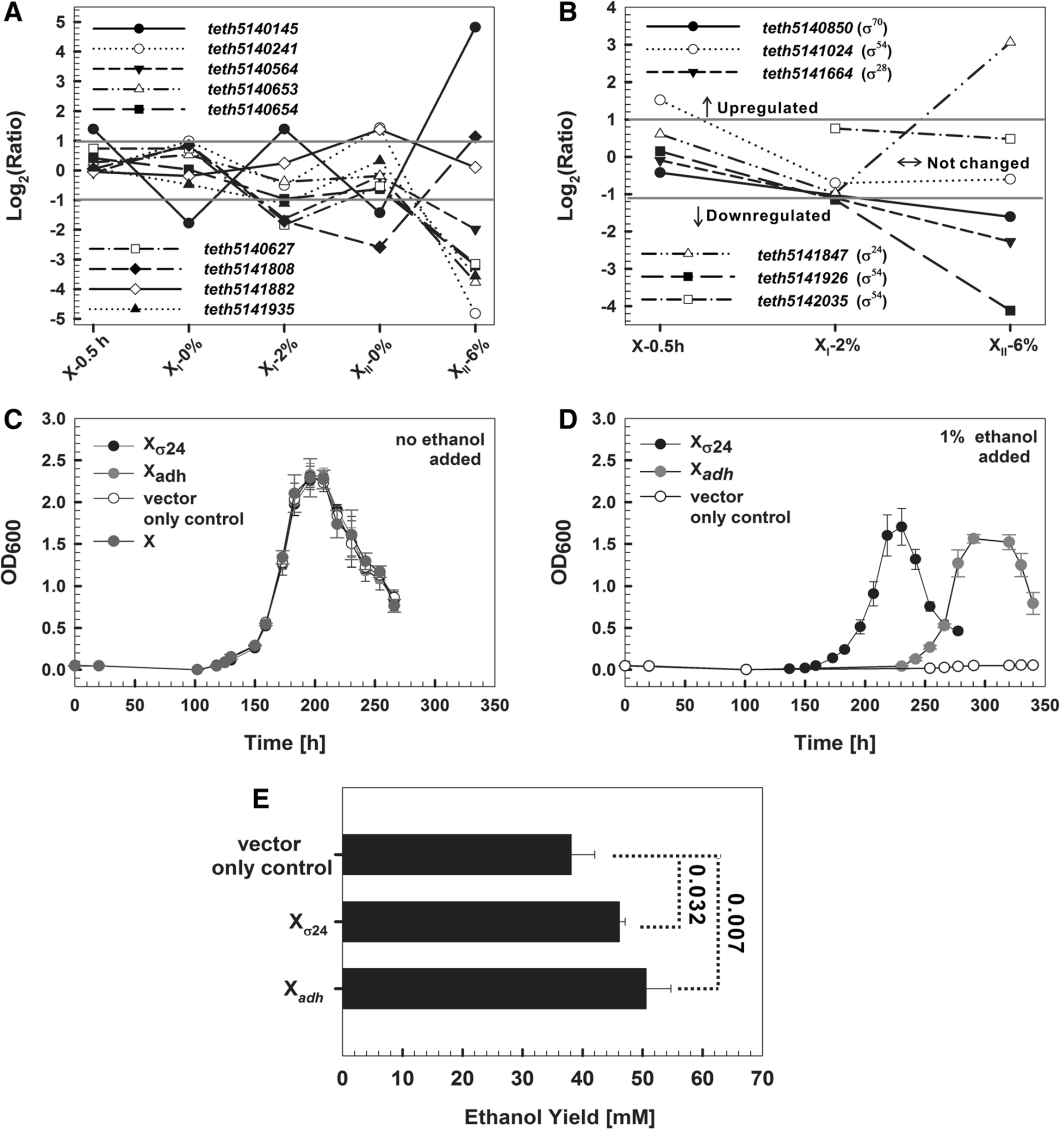
**Genetic approaches that improved both ethanol production and tolerance. (A-B)** Transcriptional programs of nine *adh*s and six ϭ factors from ethanol shock to tolerance. **(C-D)** Growth curves of *Thermoanaerobacter* sp. X514 wild-type strain and wild-type strains that carried on the pIKM1 plasmid: a vector-only control, an *adh* locus (Teth5140145-0146) or a ϭ^24^ cluster (Teth5141847-1848) under 0% **(C)** and 1% **(D)** exogenous ethanol. Strains were grown at 45^o^C in QRCM medium. **(E)** Ethanol production of these engineering strains (X_vector_, X_*adhE*_ and X_ϭ24_) at stationary phase, which were grown at 45^o^C in QRCM medium.

*First*, *adh*s were involved in both ethanol production and tolerance [4,51]; however the sheer number and apparent redundancy of this metabolic enzyme (nine such loci/operons in X514) in most bacterial genomes confounded rational engineering. In our microevolution model, an iron-containing *adh* (Teth5140145) exhibited an unique transcriptional choreography (Figure 6A): upon exposure to ethanol, its transcription was induced (by 2.6 folds) in X and X_I_ and dramatically (by 28.2 folds) induced in X_II_, yet was inhibited in X_I_ and X_II_ (which produced lower ethanol) when grown without ethanol (Additional file 2B). Such a transcriptional pattern was highly distinct from any of the other eight *adhs*, whose expression were either unaltered (8 in X, 6 in X_I_ and 2 in X_II_) or severely inhibited (2 in X_I_ and 6 in X_II_, by 3-48 folds; Figure 6A). Thus Teth5140145 might be a key junction between the co-evolving tolerance and titer. To test this hypothesis, the Teth5140145-0146 locus was cloned into a replicating plasmid pIKM1, and transformed into X. This *adh*-overexpressing strain (X_*adh*_) showed improvement in both titer and tolerance: X_*adh*_ produced 33% more ethanol than plasmid control strain X_vector_ (*p* = 0.007; Figure 6E); moreover, growth (as measured by OD_600_; Methods) under 0.25%, 0.5% and 1% exogenous ethanol were all enhanced, e.g., by 31.8 folds under 1% (Figure 6D and Additional file 21A-B), suggesting greatly improved tolerance.

Second, sigma factors are an evolutionarily conserved group of RNA-polymerase subunits that plays regulatory roles [52]. X514 harbors five types of sigma factors: ϭ^70^, ϭ^54^, ϭ^24^, ϭ^28^ and ϭ^29^. Interestingly, in our microevolution model, ϭ^24^ (Teth5141847) was unique in that it was the only ϭ factor up-regulated under 6% ethanol (Figure 6B). In *E.coli* ϭ^24^ was previously recognized as a heat-shock-specific ϭ factor [41]. Our engineered strain overexpressing Teth5141847-1848 (X_ϭ24_) showed dramatic improvement in growth, e.g., 102-fold enhancement of the control (X_vector_; as measured by OD_600_) and 26% faster than X_*adh*_ under 1%-ethanol (Figure 6D and Additional file 21A-B). Furthermore, X_ϭ24_ produced 21% higher ethanol than X_vector_ (*p* = 0.032; Figure 6E).

Finally, we found that such two co-evolving traits of ethanol productivity and ethanol tolerance can also be improved by non-genetic approaches (e.g., adaptive evolution and medium supplementation of vitamin B_12_, Additional file 21C; Part VII of Additional file 7).

## Discussion

Solvent tolerance and productivity are both crucial traits in a CBP scheme of biofuel production from cellulose, where cellulase production, cellulose hydrolysis, pentose-hexose co-utilization (one crucial feature of X514 [25]) and solvent production take place in a single bioreactor for maximal energy- and cost-efficiency [4,27,30]. Simultaneous improvement of ethanol tolerance and ethanol titer has been successfully reported in mesophiles (e.g., *S. cerevisiae* and *C. acetobutylicum*), but not yet in thermophilic bacteria [4,33]. In mesophiles such as *S. cerevisiae* and *C. acetobutylicum*, improvement in both ethanol tolerance and titer were achieved by screening strain libraries overexpressing mutant genes [14,53] or via genomic shuffling [33], yet these approaches required a set of pre determined candidate genes (e.g., two TFs, *spt15* and *taf25* were selected as the targets to generate mutation libraries by gTME [14]) or laborious mutant selection steps [14,33,53], limiting them to a narrow range of hosts (e.g., well studied model organisms). However, as all adaptation started from shock, delineating the temporal characteristics of the adaptation process and testing the mechanistic links between shock and adaptation should serve as essential foundation for modulating genome evolution, including the rational engineering of co-evolving traits.

First, our experimental model revealed the links and distinctions, both global and local, between shock response and adaptation. The “discreteness” of microevolution suggested the feasibility of phase-specific modulation of microevolution, which might carry certain advantages. This was validated by our experiments where overexpressing ϭ^24^ (a gene induced specifically at the high-tolerance phase) improved tolerance more dramatically (102 folds vs 31.8 folds) than overexpressing iron-containing *adh* (Teth5140145; a gene consistently induced along shock-to-adaptation development). Moreover, “Convergence” raised the possibility of modeling and engineering tolerance based on shock-responses. This hypothesis was validated by our experiment that overexpression of iron-containing *adh* locus (Teth5140145-0146), which were the earliest responders in shock-response, improved tolerance (Figure 6D).

Second, our study unveiled TGPA-specific features of adaptive evolution, compared to mesophiles (e.g., *E. coli* and *S. cerevisiae*). (i) The higher μ_g_ (0.045) in *Thermoanaerobacter* sp. X514 than that in *E. coli* (0.026; [5]) under solvent stress does not support the theoretically postulated much lower μ_g_ in thermophiles than in mesophiles [35]. Our finding thus tentatively suggested that TGPAs balance between the deleterious effect of the average mutation and the cost of further reducing mutation rate not by reducing μ_g_ but likely by lowering deleteriousness of mutations under stress. (ii) TGPA-specific features of ethanol-shock were unveiled, such as the most vulnerable amino acid metabolism in X514 versus the activated tryptophan biosynthesis in *S. cerevisiae*, specific activated ADI pathway (e.g., arginine deiminase) yet without activating transcription of Hsps (one key feature of the general shock-response in mesophiles [8,10]), and different mechanisms of V-type-ATPase in resisting ethanol (Figure 4B). (iii) TGPA-specific features of ethanol-tolerance were also revealed: distinct mechanisms in membrane metabolism [29,30], specific ADI pathway [38] and its related NAD/NADP octopine (Part III of Additional file 7), and a different solvent-response role of Hsps [11,13,31] (absent induction under either shock or stress; Part V of Additional file 7). Noticeably, Hsps do not seem to play a prominent role in solvent response in thermophiles and their high levels sustaining in thermophiles in the absence of stress was possibly a consequence of long-term evolution under high temperature.

Finally, by elucidating the molecular choreograph underlying an adaptive evolution under solvents, this study demonstrated a strategy to rationally identify the gene targets for engineering the tolerance-productivity relationship. In addition, our experiments showed that simultaneous improvement of ethanol tolerance and productivity is feasible in ethanogenic thermophilic bacteria, and it can be accomplished via genetic routes (e.g., metabolic enzymes (an *adh* loci; Teth5140145-0146) or transcriptional regulators (a ϭ^24^ cluster; Teth5141847-1848)). Furthermore, it is conceivable that these novel gene targets identified by our approach can serve as the foundation for rational protein-engineering or mutant protein screening to further improve ethanol tolerance and productivity in this and related thermophiles.

## Conclusions

In adaptive evolution, the molecular links between shock-response and adaptation remain poorly understood, which hinders rational engineering of solvent tolerance and correlated traits (e.g., productivity). In this study an experimental model was established to track the shock-to-adaptation microevolution in thermophiles. Under ethanol stress, the spontaneous genome mutation rate (μ_g_) in *Thermoanaerobacter*, at 0.045, appears to be equivalent to that in mesophiles (e.g. *E.coli*) [5]. Shock-response and adaptation were distinct in nature, yet both temporally phased and resilient. In the absence of stimuli, transcriptomic states of tolerance mutants resembled their stressed parental strains, while that of high-tolerance mutants resembled the extendedly shocked wild-type. Interestingly, responses to ethanol stress were phase-specific. Upon ethanol shock, X employed fructose-specific PTS, ADI pathway, Adh and a distinct mechanism of V-type ATPase. As an adaptation to ethanol, X_I_ mobilized RND efflux system and Adh, whereas X_II_, which produced higher ethanol than X_I_, employed ϭ^24^, an alcohol catabolism operon and phase-specific Hsps, modulated the operon structures of hexose/pentose transport and reinforced membrane rigidity. Exploiting these links and distinctions between shock-response and adaptation, we showed ethanol productivity and tolerance can be simultaneously improved by genetic approaches (overexpressing iron-containing *adh* or ϭ^24^).

Therefore, this study revealed thermophilic-bacteria specific features of adaptive evolution and demonstrated a rational strategy to engineer the co-evolution of industrial traits. As improvements of shock-response, stress tolerance and productivity have been crucial and shared aims in industrial applications employing thermophiles [26], our findings should be valuable not just to the production of ethanol but also to a wide variety of biofuels and biochemicals.

## Methods

### Adaptive evolution for improved ethanol tolerance

*Thermoanaerobacter* sp. X514 was cultured anaerobically in QRCM medium (1.5 g/L KH_2_PO_4_, 4.2 g/L Na_2_HPO_4_.12H_2_O, 3 g/L NaCl, 0.5 g/L NH_4_Cl, 0.2 g/L MgCl_2_, 5 g/L yeast extract, 10 g/L tryptone, 2 mg/L resazurin and 0.05g/L and Cys-HCl) supplemented with 50 mM glucose [54] at 60°C without shaking. For adaptation evolution under exogenous ethanol, sequential transfer was employed. The wild type strain (X) was initially inoculated into QRCM containing 0.5% (v/v) ethanol. When OD_600_ reached the maximum, cultures were immediately transferred into fresh 0.5%-ethanol medium on a 1:10 volume ratio. The transfer was repeated until OD_600_ reached a reproducible maximum value, cells were inoculated into 1%-ethanol medium. The cycle was repeated with increasing ethanol concentrations (until 2% ethanol) for approximately 440 generations over five months. A single clone that grew under 2% ethanol, X_I_, was isolated from the mixed cultures of mutant pools (Xp) (Figure 1B and Additional file 2A). X_I_ was mutagenized with ethyl methanesulfonate (EMS) [55] and the mutant pool screened on 6%-ethanol agar QRCM plate and subsequently in liquid QRCM (Figure 1B and Additional file 2A). One clone that grew the fastest in liquid QRCM (termed X_II_) was isolated. Further experiments confirmed that the ethanol tolerance phenotypes of both X_I_ and X_II_ were inheritable and stable after culturing for at least 60 generations in ethanol-free medium. OD_600_ improvement was measured by OD_600_ of the treatment divided by that of the control when the former reached the maximum.

In summary, X was not exposed *a priori* to exogenous ethanol and thus represented a phase of “ethanol sensitivity”, where growth was inhibited at defined medium in the mid-log with 0.15% exogenous ethanol (Additional file 1A). X_I_ represented the “low-tolerance” phase while X_II_ represented the “high-tolerance” phase, as they tolerated 2% and 6% ethanol respectively.

### Ethanol shock

To determine the effect of ethanol on X growth, a wide range of ethanol concentrations (0.1%?~?2% (v/v)) were tested first (data not shown) and then narrowed down to 0.15% (v/v) that caused stress but not significant cell death in defined medium (0.08 g/L CaCl_2_∙2H_2_O, 1.0 g/L NH_4_Cl, 0.2 g/L MgCl_2_∙6H_2_O, 1.0 g/L NaCl, 7.2 g/L HEPES, 2.52 g/L NaHCO_3_, 0.05 g/L L-cysteine-HCl, 1 ml 1000× trace element stock and 1 ml 1000× vitamin stock solution) supplemented with 50 mM glucose [8,25] (Additional file 1A). Thus all subsequent ethanol shock assays were conducted at 0.15% (v/v) ethanol in defined medium. In triplicate experiments, ethanol was added to the medium for an exogenous concentration of 0.15% when X was grown to exponential phase (OD_600_ = 0.12). An identical volume of water was added to the controls. After 0.5 h, 1 h, 2 h and 4 h of the addition, cells were harvested and cell pellets frozen immediately in liquid N_2_ and stored at -80°C prior to RNA extraction for microarray experiments.

### Ethanol stress

The mutant strains were grown at 60°C in triplicates in defined medium without ethanol (X_I_ and X_II_), with 2% ethanol (X_I_) and with 6% ethanol (X_II_) respectively. Cells were harvested at mid-exponential phase followed by microarray experiments as described above. For sugars and metabolites quantification, samples were harvested at stationary phase and analyzed using HPLC [25].

### Effects of exogenous vitamin B_12_ on ethanol production for *Thermoanaerobacter* sp. X514 (X, X_I_ and X_II_)

To test the effect of co-enzyme B_12_ on ethanol productivity, 0×, 1×, 2× to 4× B_12_ (0.1 mg/L as 1×) was added to the defined medium at 60°C, followed by inoculation of X, X_I_ and X_II_, respectively. Ethanol concentration at stationary phase was measured by HPLC [25].

### Microarray experiments and data analysis

*Thermoanaerobacter* sp. X514 whole-genome oligonucleotide (70mer) microarray [25] was used in this study. Total cellular RNA and genomic DNA were isolated, labeled and then hybridization and data analysis performed as previously described [25]. For ethanol shock, the trasncriptomes of X-0.15% (X cells cultured at defined medium with 0.15% exogenous ethanol; Additional file 1A) were compared to that of X-0% (X cells cultured without exogenous ethanol). On the other hand, the transcriptomes of X_I_ under 0% and 2% ethanol and those of X_II_ under 0% and 6% ethanol were compared respectively to reveal the mechanism of ethanol adaptation. Cutoffs of mean |log_2_ (R_treatment_/R_control_)| ≥1.0 and |Z score|?≥?2.0 were used to determine significant expression changes [25,34]. The totally 39 microarray datasets were deposited as NCBI GEOGSE32630.

### Gene co-expression networks for shock response

Twelve microarray datasets for the ethanol shock (three replicates for each of the four time points) and the corresponding twelve control datasets were respectively generated and used via co-expression analysis to construct the two co-expression networks with random matrix theory approach [25,56] (Additional file 6). For each spot on microarray, a normalized Cy5/Cy3 ratio (R) was calculated and its logarithmic transformation performed. Pearson correlation coefficient cut-off was 0.98 (both for ES^+^ and ES^-^) between each gene-pair. The modules were separated by fast greedy modularity optimization [56]. Hierarchical clustering analysis was performed using K-Means/K-Medians Clustering to identify expression patterns each shared by a sub-set of genes throughout the duration of the ethanol shock response (Additional file 9).

### Hierarchical clustering analysis of transcriptomes

Hierarchical clustering analysis (support tress) among the nine transcriptional profiles from shock (X-0.15% at each of the four time points of 0.5 h, 1 h, 2 h and 4 h) to adaptation (X_I_-0%, X_I_-2%, X_II_-0%, X_II_-6%, each in triplicates) was performed with TM4 software [57] based on Pearson Correlation. Similarly, the clustering analysis among the eight “relative” transcriptomic changes (using genes with more than 2 fold changes) from shock (X-0.15% vs X-0% at each of the four time points of 0.5 h, 1 h, 2 h and 4 h) to adaptation (X_I_-0% vs X-0%, X_I_-2% vs X_I_-0%, X_II_-0% vs X-0%, X_II_-6% vs X_II_-0%, each in triplicates) was performed via the same method.

### RNA-Seq for detecting structural variation of transcripts

For X, X_I_ and X_II_, 10 μg of the same total RNA samples from ethanol stress and normal growth condition (in the absence of ethanol) in triplicates were used for high-throughput RNA-Sequencing. The cDNA libraries (X-0%, X_I_-0%, X_I_-2%, X_II_-0% and X_II_-6%) were constructed as previously described [58]. The samples were quantified spectrophotometrically using Nanodrop (Thermo, USA) and sequenced in a Solexa GA-IIx (Illumina, USA). The raw 2 × 100bp reads, after quality screening, were mapped to the *Thermoanaerobacter* sp. X514 reference genome sequence [34] (NCBI accession number: NC_010320.1) using SOAP [59], allowing for 2nt mismatches. These uniquely mapped sequences were further analyzed to calculate transcript coverage map based on the number of uniquely mapped reads per locus. The RNA-Seq datasets were deposited as NCBI accession number SRA046273.1.

### Detection of dynamic operon structures

Genes within an operon were defined based on continuous read coverage, transcript abundance and detection of pair-end reads among these genes in all of the triplicates. A new operon was defined when two requirements were met in each of the triplicate samples: 1) uniquely mapped pair-end reads were detected; 2) a significant change (greater than two-fold) of read coverage was found between genes in one predicted polycistron.

### Genome-wide mutation profiling via whole-genome sequencing

Genomic DNA of X, Xp, X_I_ and X_II_ were isolated [25] and then shot gun libraries constructed [58] and sequenced on Solexa GA-IIx (Illumina, USA). MAQ [60], Samtools [61] and GATK [62] were used respectively for read alignment to *Thermoanaerobacter* sp. X514 reference genome (NC_010320.1; [34]) and SNP calling. Those predicted SNPs shared among the three were then manually examined (Additional file 10) and then validated by sequencing a selected set of mutated genes (five in X_I_ and six in X_II_) using gene-specific primer pairs (Additional file 12A). For each gene, ten clones were randomly picked for Sanger sequencing (Invitrogen, USA). The results were consistent with Solexa sequencing (Additional file 12B). All sequences were deposited under NCBI accession number SRA046273.1.

### Plasmid and strain construction

A 3.4 kb PCR fragment encoding the 3.1 kb iron-containing *adh* and NADH oxidase gene (Teth5140145-0146) flanked by its 197 bp promoter and 48 bp transcription terminator region was amplified from the genome of *Thermoanaerobacter* sp. X514 and subsequently ligated into plasmid pIKM1 [54] through *Eco*RI and *Bam*HI. Similarly, 1.7 kb PCR fragment encoding the 1.4 kb ϭ^24^ factor and hypothetical protein gene (Teth5141847-1848) with its 245 bp promoter and 80 bp transcription terminator region was amplified and subcloned into pIKM1 through *Xba*I and *Kpn*I. Plasmids were then transformed into *Thermoanaerobacter* sp. X514 wild type strain (X) based on our published protocol [54]. These plasmid containing strains were cultured in QRCM medium at 45°C and cell samples were collected at stationary phase for ethanol titer quantification.

### Homology modeling of the structure of mutant proteins

For the X514 wild-type and mutant protein sequences (DeoR family transcriptional factor (Teth5141305) and the ADH domain of AdhE (Teth5140627), queries were aligned to structural templates in PDB using NCBI protein BLAST. The homology models of 3D protein structures that included cofactors and ligands were constructed via MODELLER [63].

## Abbreviations

AK: Acetate kinase; Aldh (Teth5141942): Aldehyde dehydrogenase; Adh (Teth5140145): Alcohol dehydrogenase; ADI pathway (Teth5140483-0485): Arginine deiminase pathway; AdhE (Teth5140627): Bifunctional aldehyde/alcohol dehydrogenase; Cas immunity system: CRISPRs-associated immunity system; Euts (Teth5141943-1946): Ethanolamine utilization protein genes; XII: 6% ethanol tolerant mutant; EMS: Ethyl methanesulfonate; μg: Genome mutation rate; Hsps: Heat shock proteins; ipmdh: 3-isopropylmalate dehydrogenase; Ldh (Teth5140216): Lactate dehydrogenase; XI: One single colony from Xp; Oad (Teth5141582): Oxaloacetate decarboxylase; PTS: Phosphotransferase system; Xp: Pool of 2% ethanol tolerant spontaneous mutants of *Thermoanaerobacter* sp. X514; MreC (Teth5142133): Rod shape-determining protein; RodA (Teth5142127): Rod shape-determining protein; RND efflux system: Resistance-nodulation-cell division family efflux transporter; TetR: Tetracycline repressor family transcription factor; TGPAs: Thermophilic gram-positive anaerobes; X: Wild-type *Thermoanaerobacter* sp. X514.

## Competing interests

The authors declare that they have no conflict of interest.

## Authors’ contributions

LL and JX conceived and designed the study. LL, YJ, HS, XZ, LT and KW performed experiments. JZ and ZH assisted microarray experiment design and analysis. QT constructed co-expression gene networks. RH and QZ assisted RNA-Seq data analysis. YL and QC assisted protein structure modeling. JX and LL analyzed all data and wrote the paper. All authors read and approved the final manuscript.

## Supplementary Material

Additional file 1 **Impact of ethanol shock on the growth and gene expression of *Thermoanaerobacter *sp. X514 (wild type). A**) Ethanol of different concentrations was added to cultures in early-mid exponential phase, and growth was subsequently monitored as OD_600_. Data were averaged from triplicate cultures, with error bars indicating standard deviations. **B**) Numbers of the significantly up- or down-regulated genes under each condition. Each column represented the number of genes with significant expression changes (|log_2_*R*|?≥?1 and |Z score| ≥2|) at the corresponding time points. Click here for file

Additional file 2 **Thermoanaerobic growth conditions and glucose fermentation by *Thermoanaerobacter *sp. X514 wild type and ethanol tolerant mutants. A**) Growth curves of X, X_I_ and X_II_ in defined medium with or without exogenous ethanol. **B**) Substrate utilization and product profiles of X, X_I_ and X_II_ in defined medium. Click here for file

Additional file 3 **Scanning electron microscopy images of the *Thermoanaerobacter *sp. X514 wild-type and mutants.** Click here for file

Additional file 4 **Statistical analysis of the mutations in X, Xp, X**_I _**and X**_**II **_**Genomes.** Click here for file

Additional file 5 **Functional patterns of significantly expression-altered Genes in X**_**I **_**(A) and X**_**II **_**(B) under ethanol-supplemented and ethanol-free media respectively.** Proportions of differentially expressed genes under each COG among the total number of differentially expressed genes were indicated. Click here for file

Additional file 6 **Gene co-expression networks of *Thermoanaerobacter *under ethanol shock and normal growth condition. A**) A global view of the network under normal growth condition (ES^-^). **B**) A global view of the network under ethanol shock (ES^+^). Number in bracket represents the number of genes in each module. Each node represented a gene, which was color-coded using its predicted COG-based functional classification (http://www.ncbi.nlm.nih.gov/nuccore/NC_010320; NC_010320.1). Click here for file

Additional file 7 Supplemental Materials. Click here for file

Additional file 8 The List of differentially expressed genes by X for at least one time points in each cluster under 0.15% (v/v) ethanol shock. Click here for file

Additional file 9 **Clustering results of gene-expression patterns in the X under ethanol shock.** Ten clusters were obtained by the HCL method (TM4 software). Each panel displayed the expression dynamics of one such cluster. The horizontal axis indicated the time points of the data, and vertical axis was log (base 2) expression ratio. Click here for file

Additional file 10 **Genomic mutations shared among the three mutations-lists identified by MAQ, Samtools and GATK respectively. A**) X; **B**) Xp; **C**) X_I_; **D**) X_II_. Click here for file

Additional file 11 **The SNPs in non-coding region.** Red: the mutated base; base in brackets: reference base at mutated point; dash: single base deletion. Promoters were predicted by Berkeley Drosophila Genome Project (prokaryote organism; http://www.fruitfly.org/seq_tools/promoter.html). Click here for file

Additional file 12 **Experimental validation of SNPs in X**_**I **_**and X**_**II**_**. A**) The primer sequences used for PCR and then Sanger sequencing. **B**) The SNPs validated by Sanger sequencing. Click here for file

Additional file 13 **Priori ethanol stress rewired cellular networks (X**_**I **_**and X**_**II**_**).** Key transport, metabolic and regulatory genes and pathways were illustrated. OD: NAD/NADP octopine dehydrogenase; FITP: ferrous iron transport protein B. Other abbreviations were as in Figure 5. Click here for file

Additional file 14 **Developmental model of solvent-tolerance traits in thermopiles. A**) Model of cellular responses upon environmental stimuli: the transition from shock response (wild type) to low-tolerance (X_I_) and eventually to high-tolerance (X_II_). **B**) Model of the cellular states after *a priori* long-term exposure to exogenous ethanol. Boxes represented genes/pathways, whereas box size indicated the number of differentially expressed genes in the pathway. Differential-expression ratios (log_2_*R*) for the genes/pathways were represented by colors. Asterisks indicated non-synonymous mutated genes in the pathways or the SNPs in predicted promoters of genes. Tra (P): ABC transporter related in COG P; MM: membrane metabolism; AAM: amino acid metabolism; LFA: long-chain fatty acid. Click here for file

Additional file 15 **Differentially expressed genes in mutant. A**) X_I_ under normal growth condition (X_I_-0%). **B**) X_II_ under normal growth condition (X_II_-0%). Click here for file

Additional file 16 **Differentially expressed genes in mutants under ethanol stress. A**) X_I_ under 2% ethanol (X_I_-2%).** B**) X_II_ under 6% ethanol (X_II_-6%). Click here for file

Additional file 17 **Homology models of the mutant protein structures DeoR (A) and AdhE (B) in *Thermoanaerobacter *sp. X514.** Mutation sites, cofactors and ligands were respectively labeled. Click here for file

Additional file 18 **The Dynamic operons between X and X**_**II **_**(Teth5140597-0601).** Gray and black areas respectively represented the coverage of mRNA in X and X_II_ in ethanol-free media. The scale with the relative score indicated, for each library and at a given nucleotide (among the three biological replicates), the mean number of uniquely mapped reads normalized to the total number of such reads. Click here for file

Additional file 19 **The Dynamic operons between X**_**I **_**and X**_**II **_**in the absence of ethanol. A**-**B**) Teth5140161-0166; C-D) Teth5140412-0414; **E**-**F**) Teth5141359-1361. The scale was as in Additional file 18. Click here for file

Additional file 20 **The Dynamic operon between X**_**II**_**-0% and X**_**II**_**-6% (Teth5141785-1787).** The scale was as in Additional file 18. Click here for file

Additional file 21 **Genetic and non-genetic approaches that improved both ethanol production and tolerance.A**-**B**) Growth curves of *Thermoanaerobacter* sp. X514 wild type strains that carried different plasmids (a vector-only control, an *adh* cluster (Teth5140145-0146) or a ϭ^24^ cluster (Teth5141847-1848)). Strains were grown at 45°C in QRCM medium with 0.25% and 0.5% added ethanol respectively. **C**) Fermentation experiments testing effects of vitamin B_12_ on ethanol production of tolerant mutants in defined medium at 60°C. Vitamin B_12_ concentrations in defined medium were indicated on x-axis. The means (and standard deviation) for the three biological replicates were shown for each sample. Differences in means were evaluated using one-tailed paired *t*-test (those with *p*<0.05 were shown). Click here for file
