# Supplementary figures and images for: Microevolution from shock to adaptation revealed strategies improving ethanol tolerance and production in Thermoanaerobacter
Source: Biotechnol Biofuels. 2013 Jul 22;6:103. doi: 10.1186/1754-6834-6-103 (PMC3751872; doi:10.1186/1754-6834-6-103)

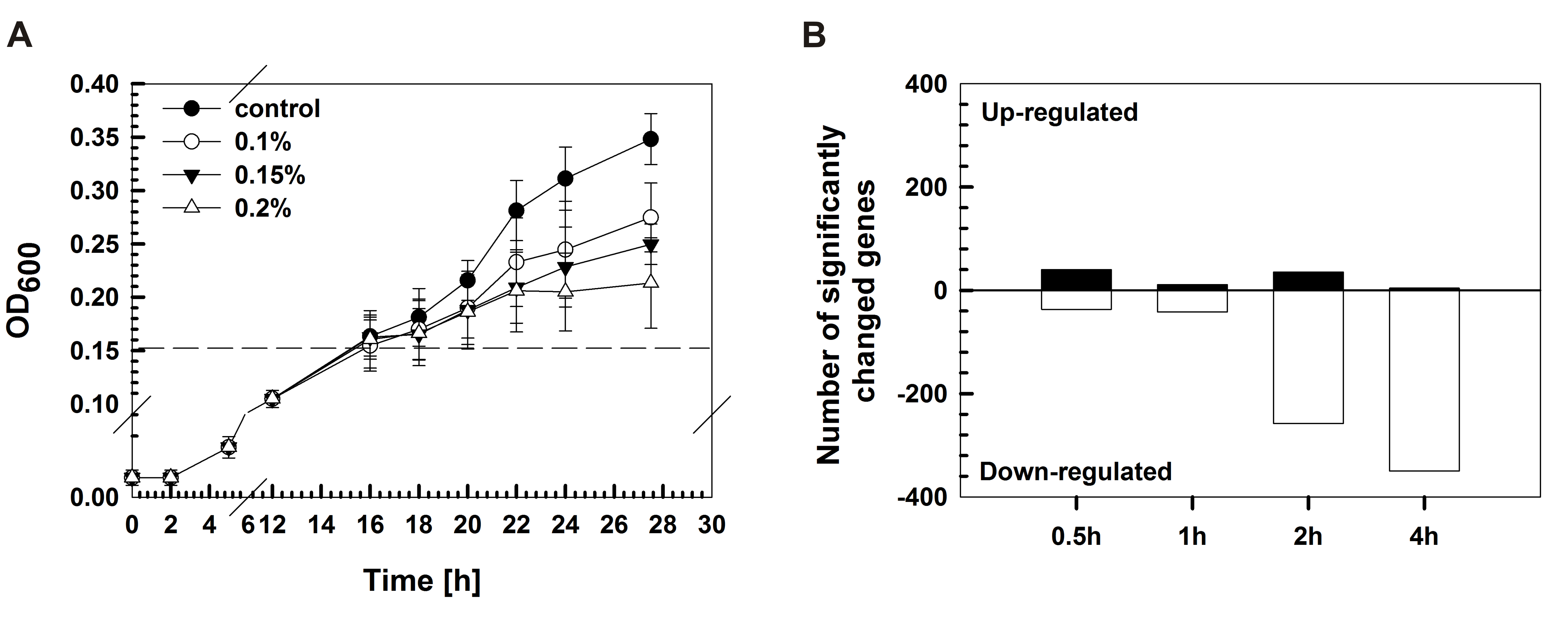

Supplement: Additional file 1 — Impact of ethanol shock on the growth and gene expression of Thermoanaerobacter sp. X514 (wild type). A) Ethanol of different concentrations was added to cultures in early-mid exponential phase, and growth was subsequently monitored as OD600. Data were averaged from triplicate cultures, with error bars indicating standard deviations. B) Numbers of the significantly up- or down-regulated genes under each condition. Each column represented the number of genes with significant expression changes (|log2R|?≥?1 and |Z score| ≥2|) at the corresponding time points. [file 1754-6834-6-103-S1.tiff]

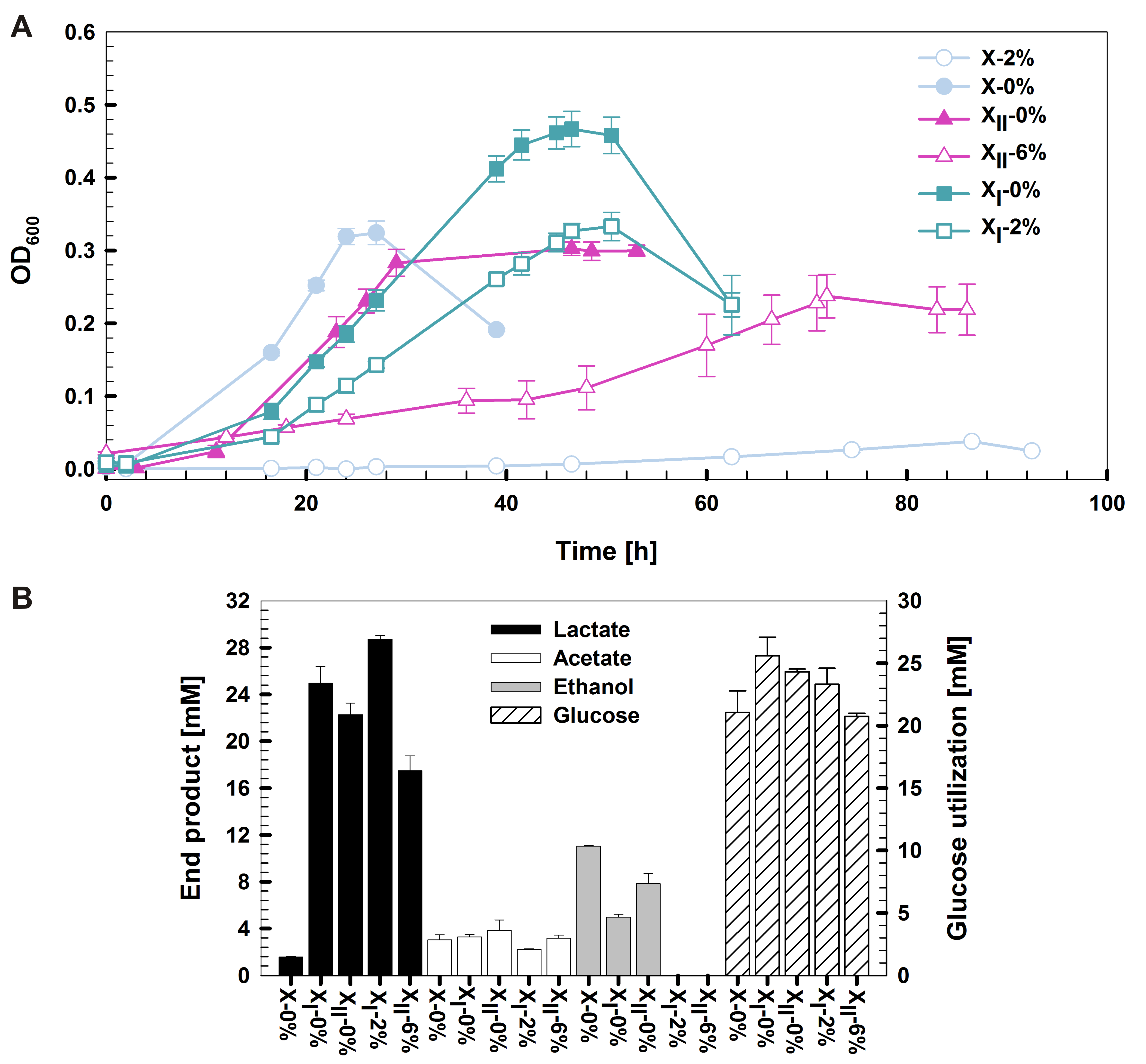

Supplement: Additional file 2 — Thermoanaerobic growth conditions and glucose fermentation by Thermoanaerobacter sp. X514 wild type and ethanol tolerant mutants. A) Growth curves of X, XI and XII in defined medium with or without exogenous ethanol. B) Substrate utilization and product profiles of X, XI and XII in defined medium. [file 1754-6834-6-103-S2.tiff]

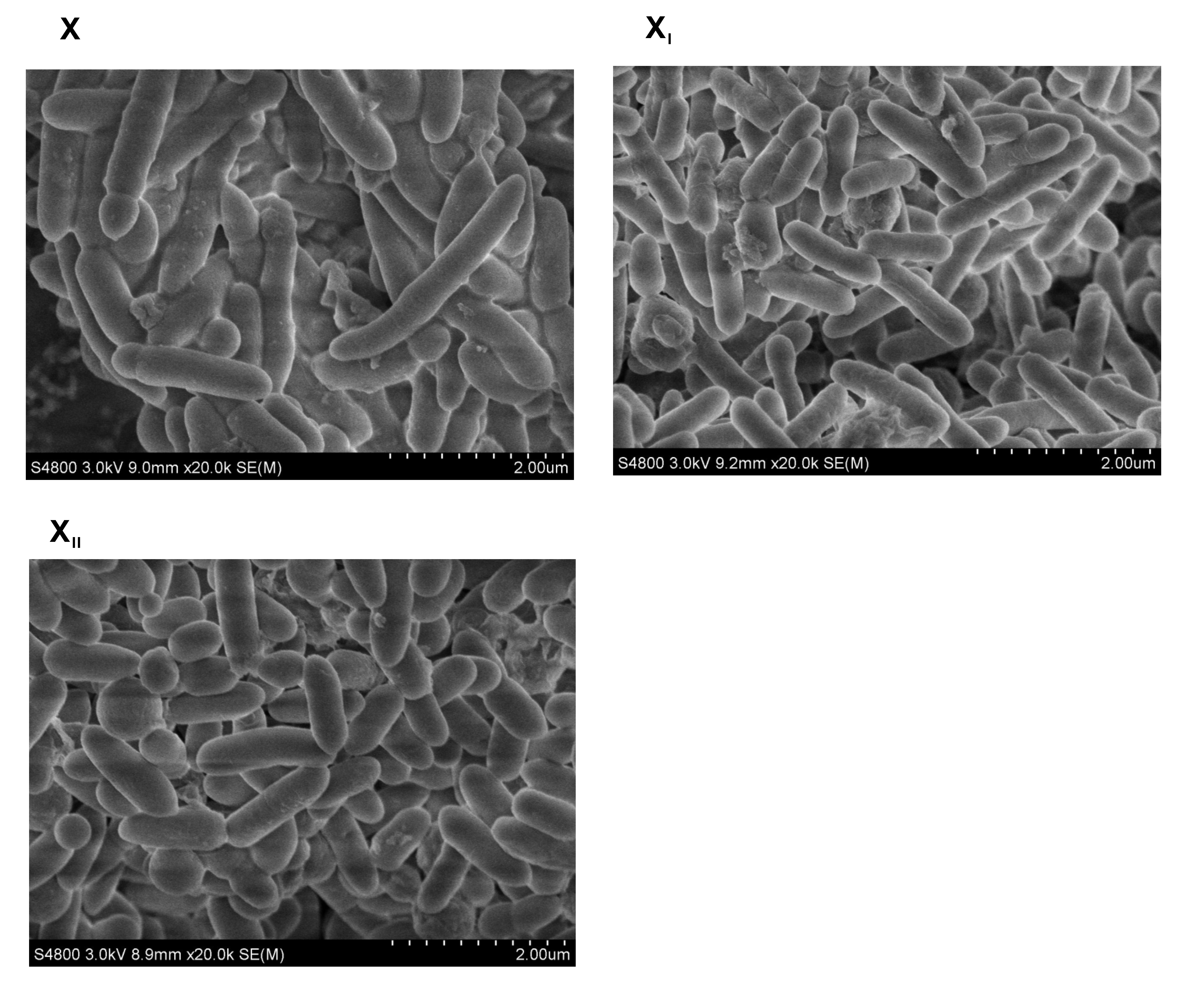

Supplement: Additional file 3 — Scanning electron microscopy images of the Thermoanaerobacter sp. X514 wild-type and mutants. [file 1754-6834-6-103-S3.tiff]

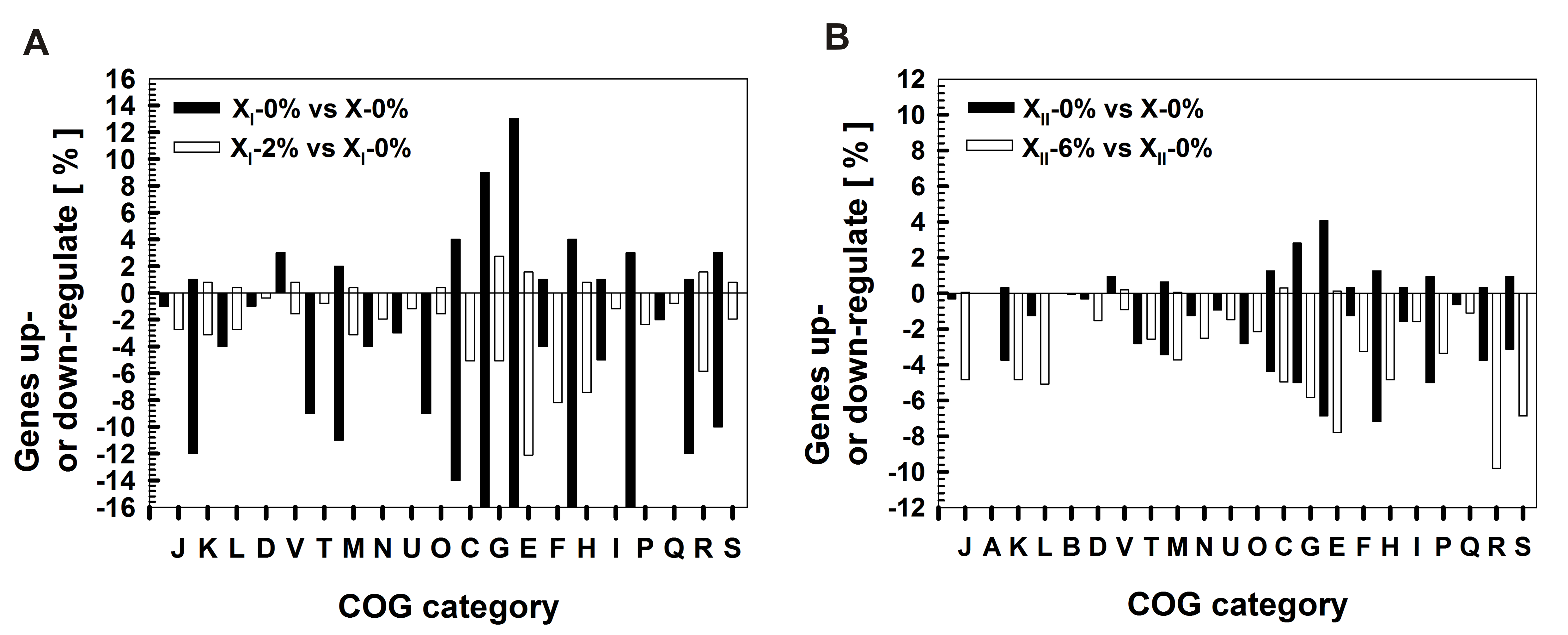

Supplement: Additional file 5 — Functional patterns of significantly expression-altered Genes in XI (A) and XII (B) under ethanol-supplemented and ethanol-free media respectively. Proportions of differentially expressed genes under each COG among the total number of differentially expressed genes were indicated. [file 1754-6834-6-103-S5.tiff]

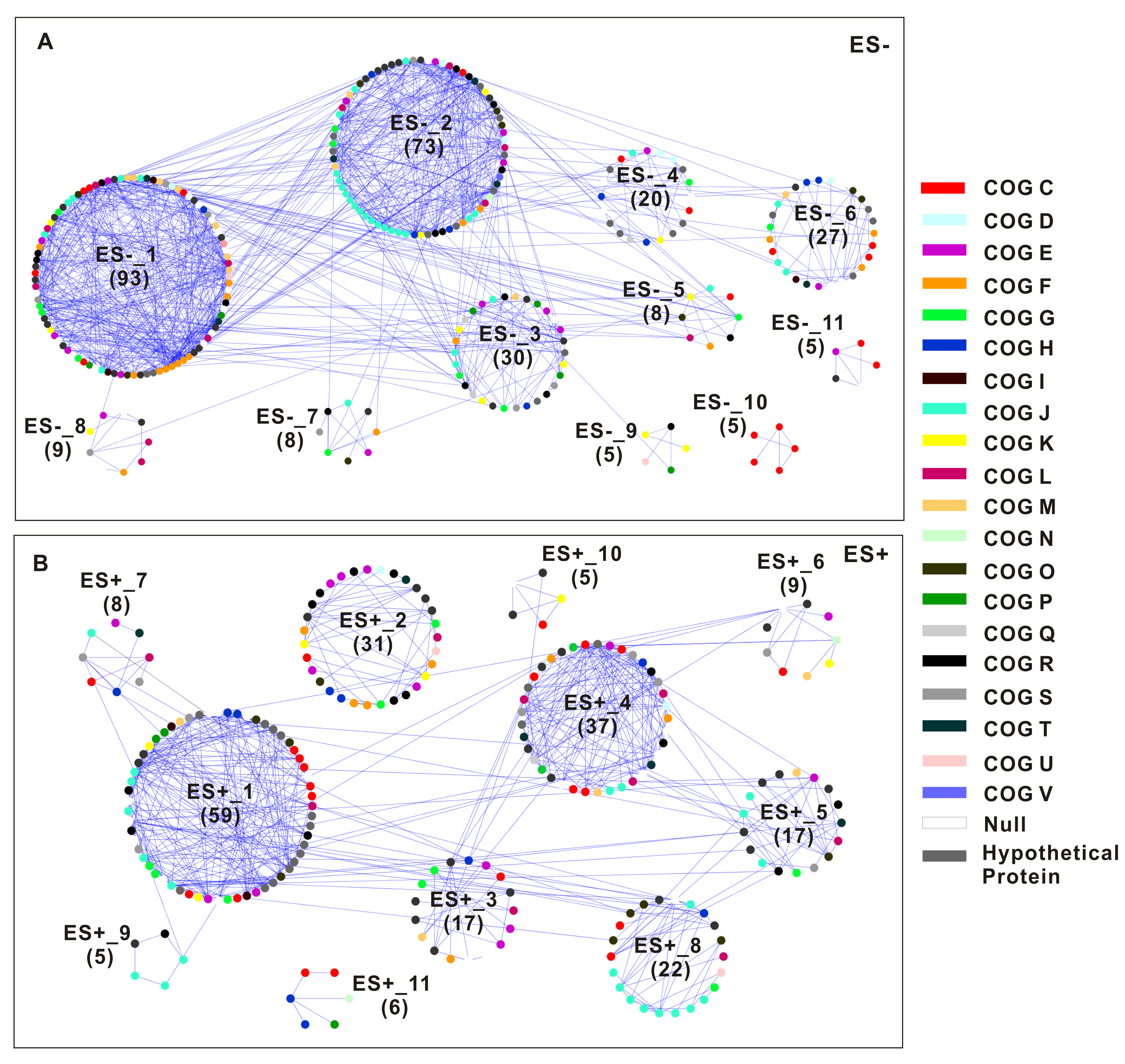

Supplement: Additional file 6 — Gene co-expression networks of Thermoanaerobacter under ethanol shock and normal growth condition. A) A global view of the network under normal growth condition (ES-). B) A global view of the network under ethanol shock (ES+). Number in bracket represents the number of genes in each module. Each node represented a gene, which was color-coded using its predicted COG-based functional classification (http://www.ncbi.nlm.nih.gov/nuccore/NC_010320; NC_010320.1). [file 1754-6834-6-103-S6.tiff]

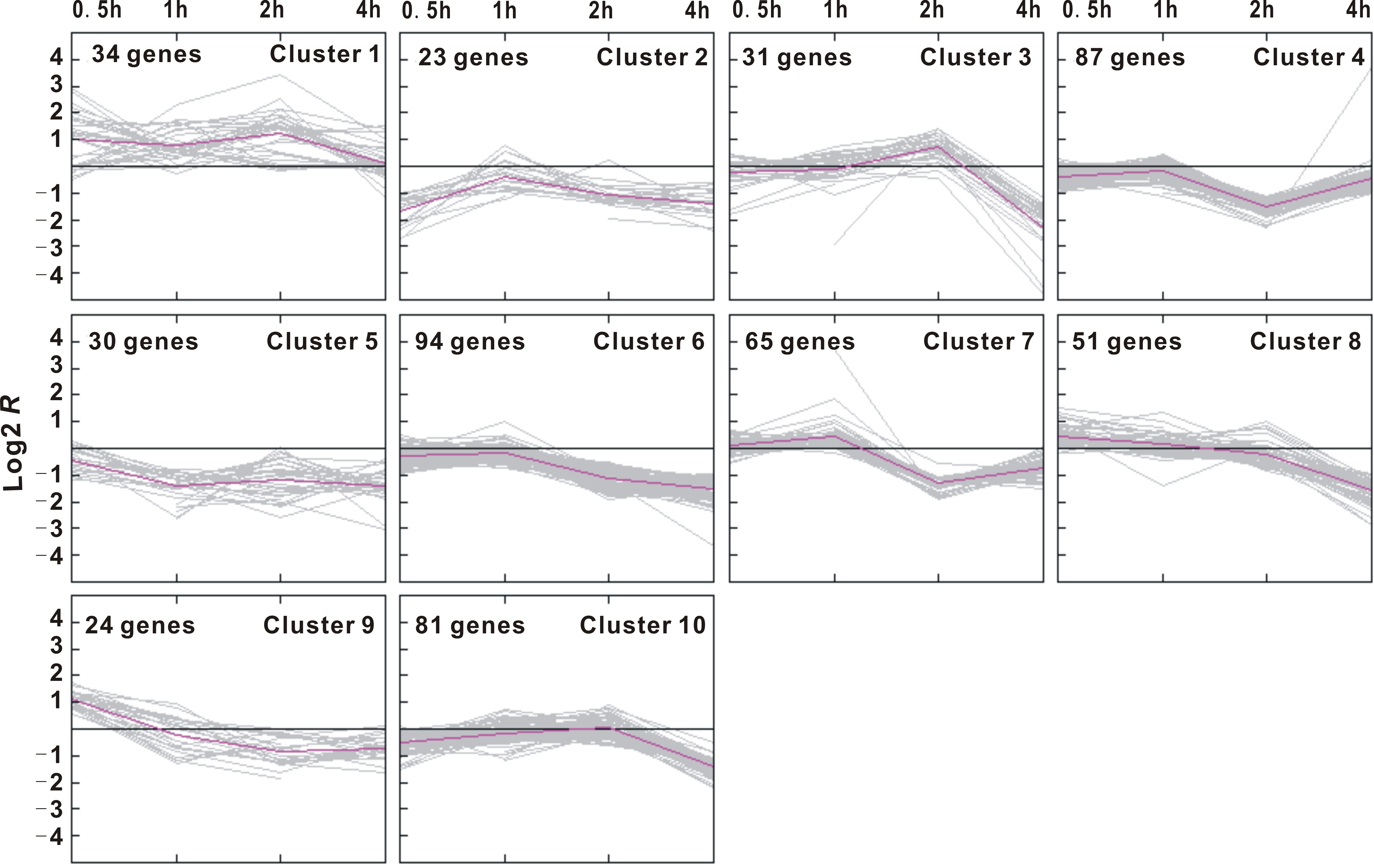

Supplement: Additional file 9 — Clustering results of gene-expression patterns in the X under ethanol shock. Ten clusters were obtained by the HCL method (TM4 software). Each panel displayed the expression dynamics of one such cluster. The horizontal axis indicated the time points of the data, and vertical axis was log (base 2) expression ratio. [file 1754-6834-6-103-S9.tiff]

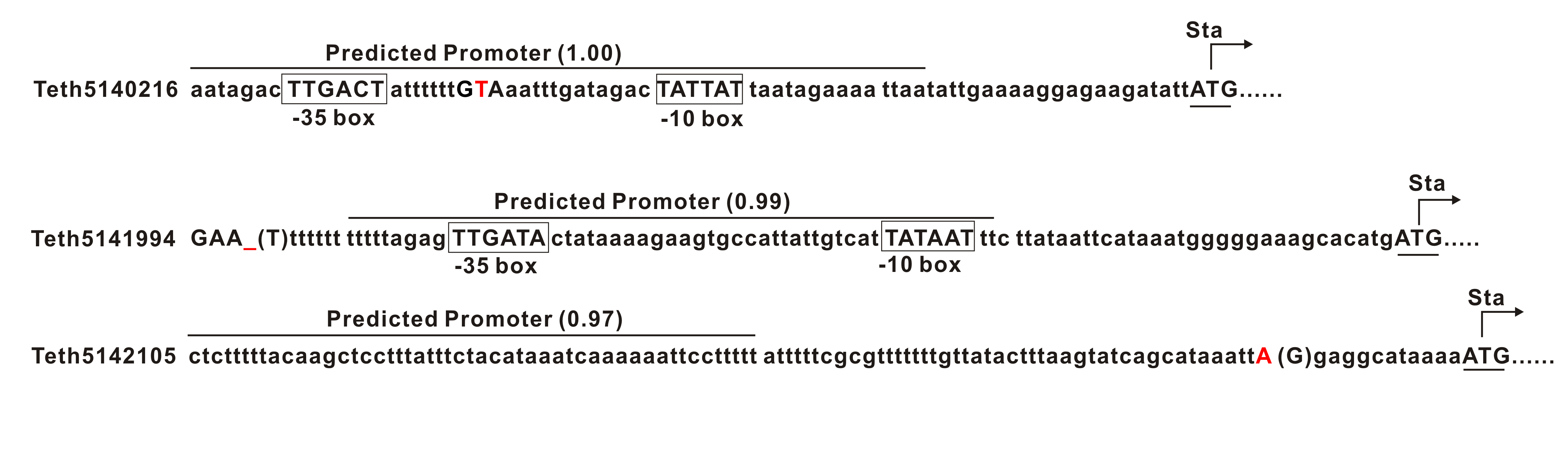

Supplement: Additional file 11 — The SNPs in non-coding region. Red: the mutated base; base in brackets: reference base at mutated point; dash: single base deletion. Promoters were predicted by Berkeley Drosophila Genome Project (prokaryote organism; http://www.fruitfly.org/seq_tools/promoter.html). [file 1754-6834-6-103-S11.tiff]

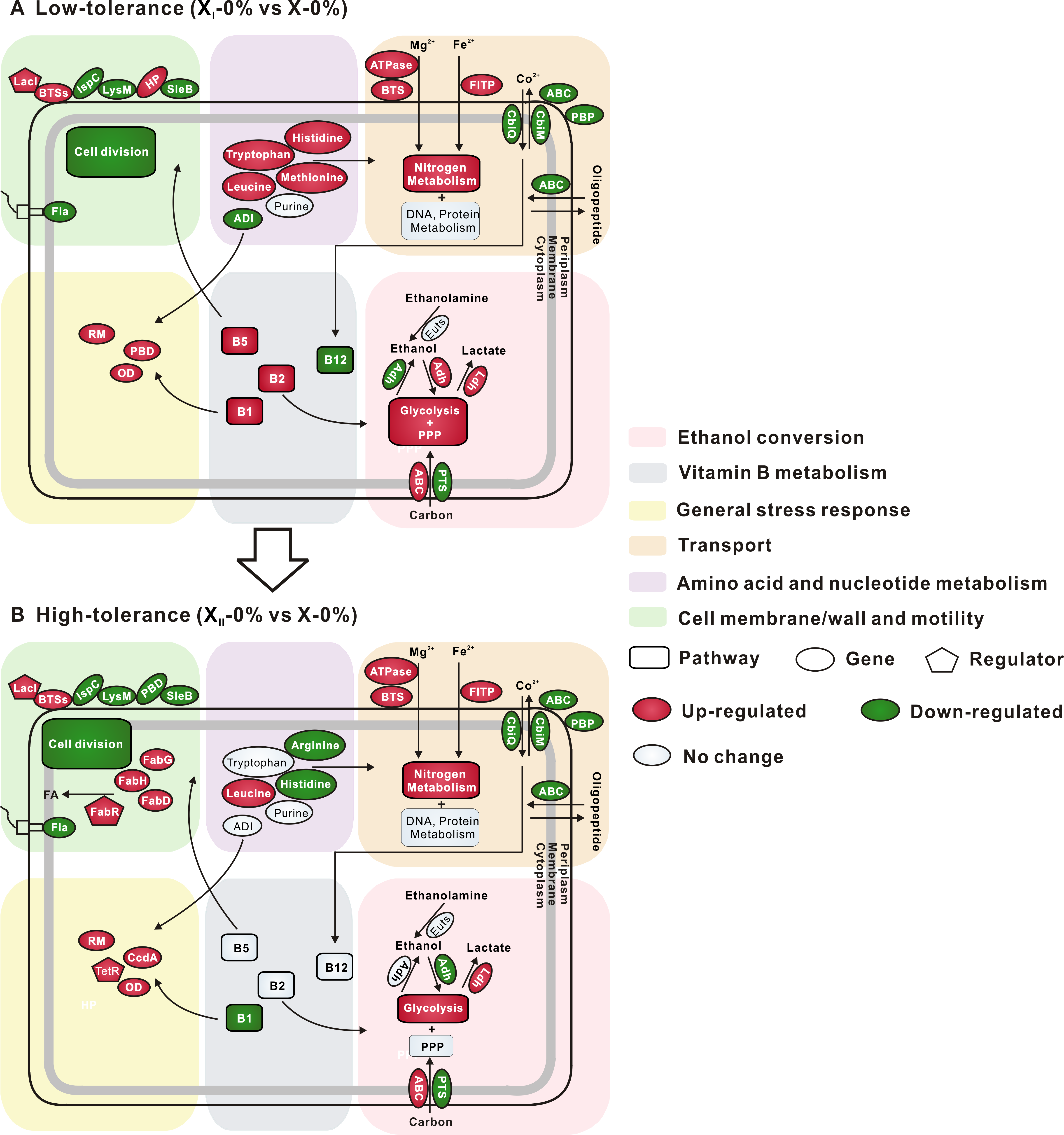

Supplement: Additional file 13 — Priori ethanol stress rewired cellular networks (XI and XII). Key transport, metabolic and regulatory genes and pathways were illustrated. OD: NAD/NADP octopine dehydrogenase; FITP: ferrous iron transport protein B. Other abbreviations were as in Figure 5. [file 1754-6834-6-103-S13.tiff]

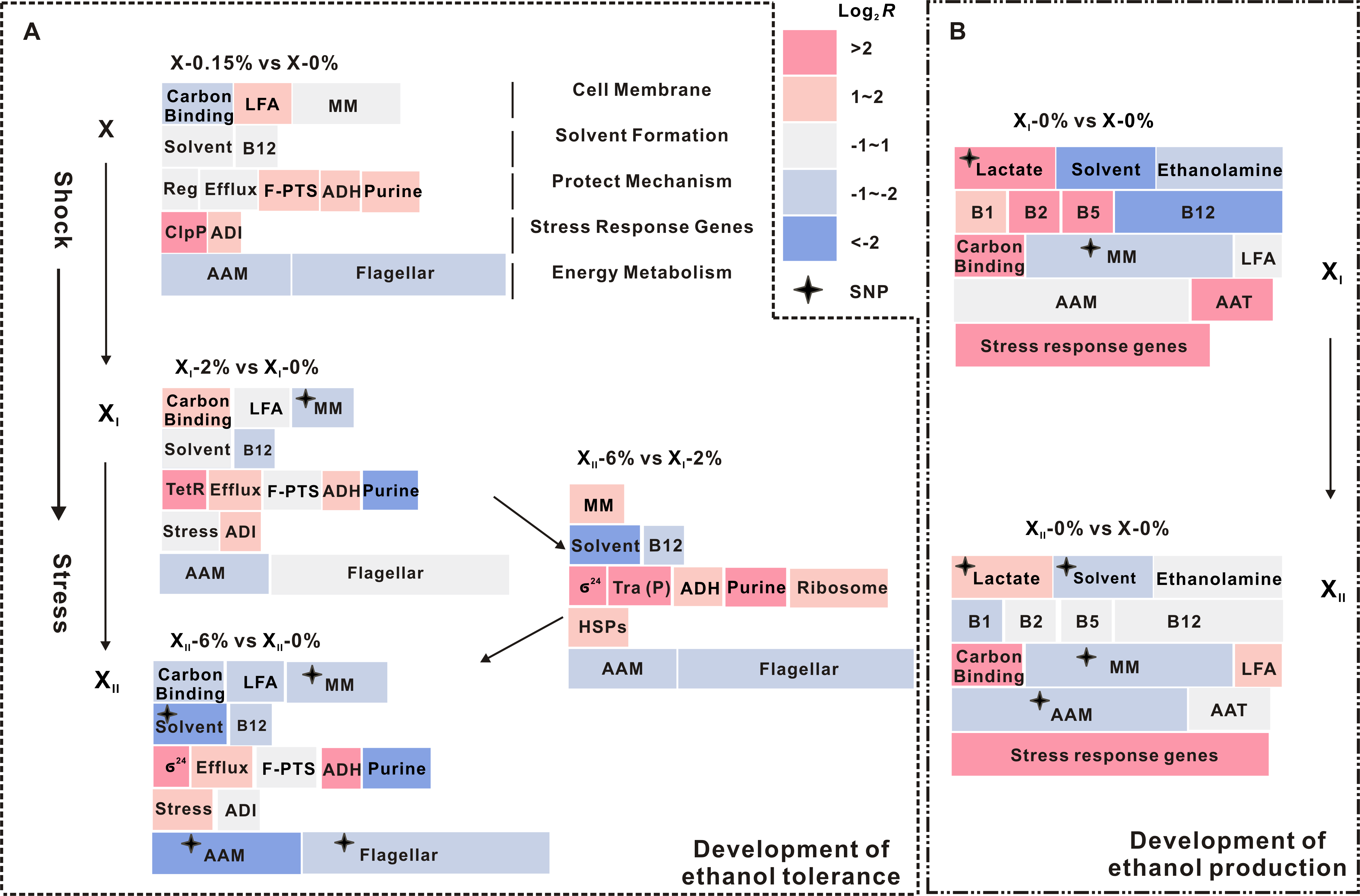

Supplement: Additional file 14 — Developmental model of solvent-tolerance traits in thermopiles. A) Model of cellular responses upon environmental stimuli: the transition from shock response (wild type) to low-tolerance (XI) and eventually to high-tolerance (XII). B) Model of the cellular states after a priori long-term exposure to exogenous ethanol. Boxes represented genes/pathways, whereas box size indicated the number of differentially expressed genes in the pathway. Differential-expression ratios (log2R) for the genes/pathways were represented by colors. Asterisks indicated non-synonymous mutated genes in the pathways or the SNPs in predicted promoters of genes. Tra (P): ABC transporter related in COG P; MM: membrane metabolism; AAM: amino acid metabolism; LFA: long-chain fatty acid. [file 1754-6834-6-103-S14.tiff]

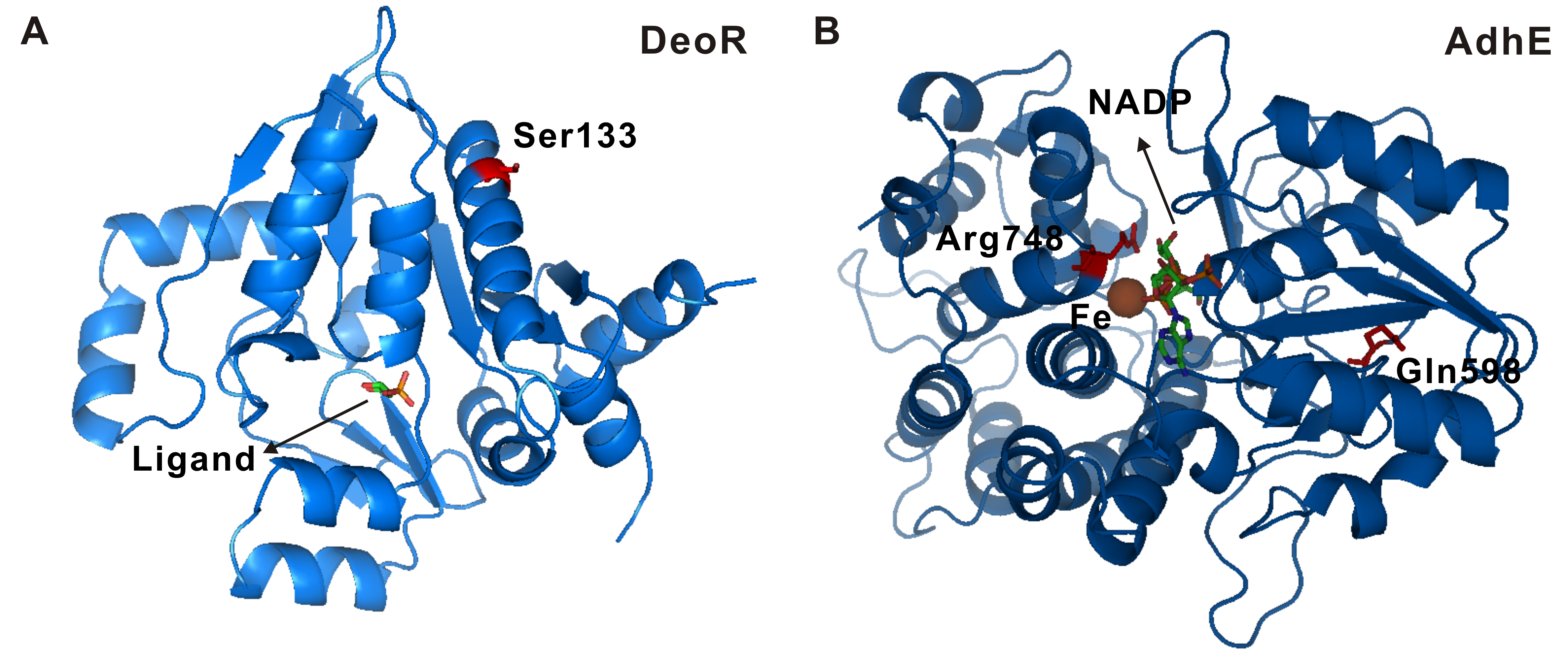

Supplement: Additional file 17 — Homology models of the mutant protein structures DeoR (A) and AdhE (B) in Thermoanaerobacter sp. X514. Mutation sites, cofactors and ligands were respectively labeled. [file 1754-6834-6-103-S17.tiff]

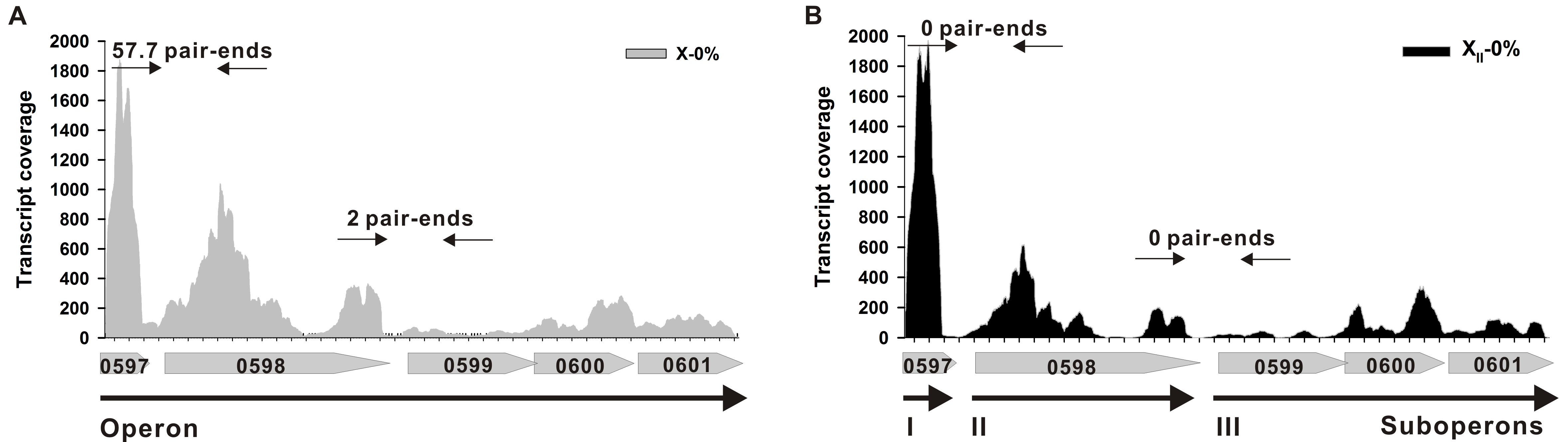

Supplement: Additional file 18 — The Dynamic operons between X and XII (Teth5140597-0601). Gray and black areas respectively represented the coverage of mRNA in X and XII in ethanol-free media. The scale with the relative score indicated, for each library and at a given nucleotide (among the three biological replicates), the mean number of uniquely mapped reads normalized to the total number of such reads. [file 1754-6834-6-103-S18.tiff]

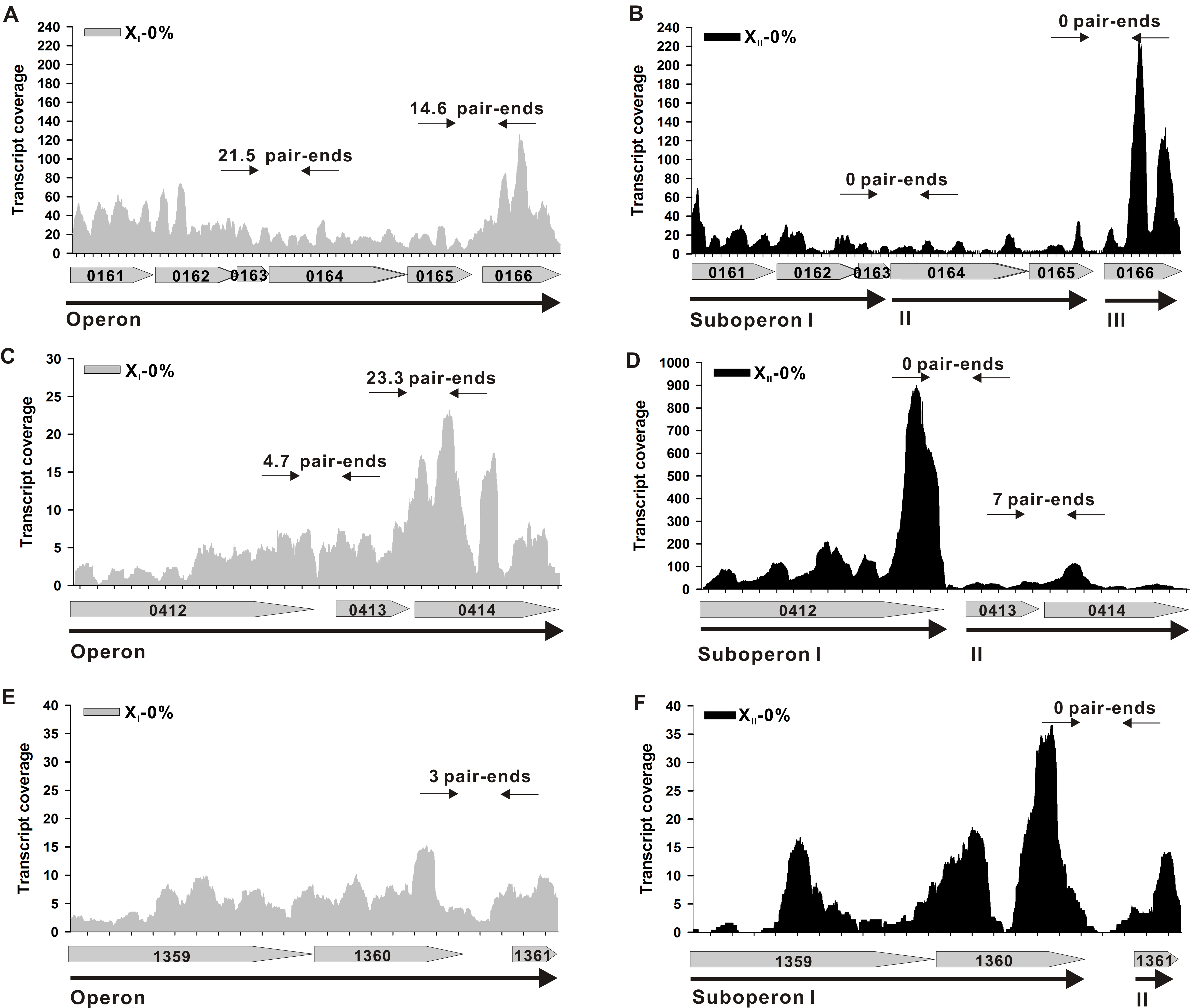

Supplement: Additional file 19 — The Dynamic operons between XI and XII in the absence of ethanol. A-B) Teth5140161-0166; C-D) Teth5140412-0414; E-F) Teth5141359-1361. The scale was as in Additional file 18. [file 1754-6834-6-103-S19.tiff]

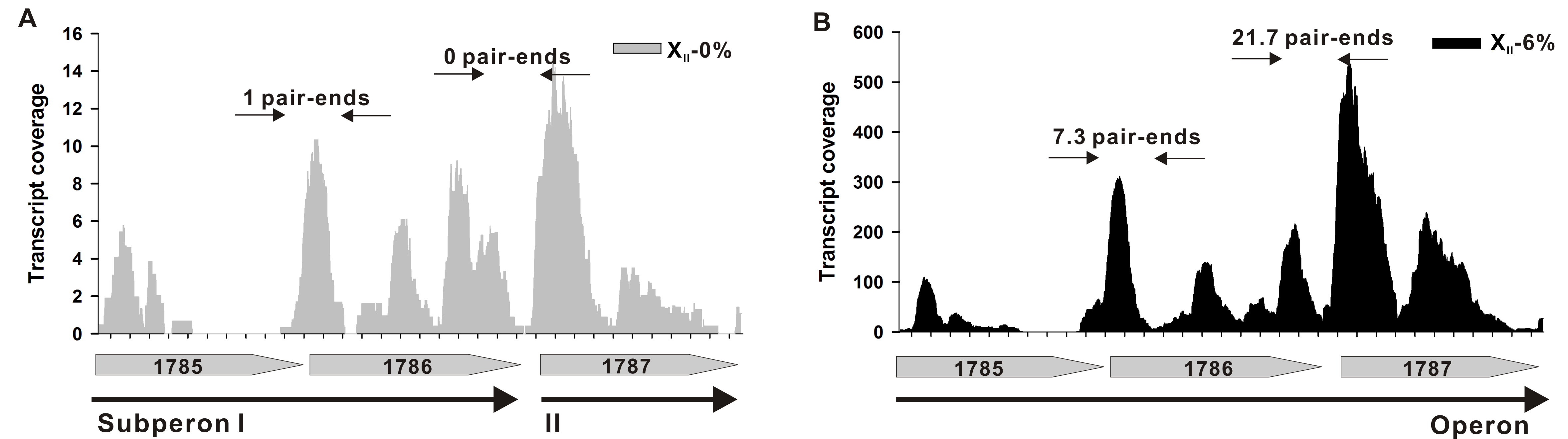

Supplement: Additional file 20 — The Dynamic operon between XII-0% and XII-6% (Teth5141785-1787). The scale was as in Additional file 18. [file 1754-6834-6-103-S20.tiff]

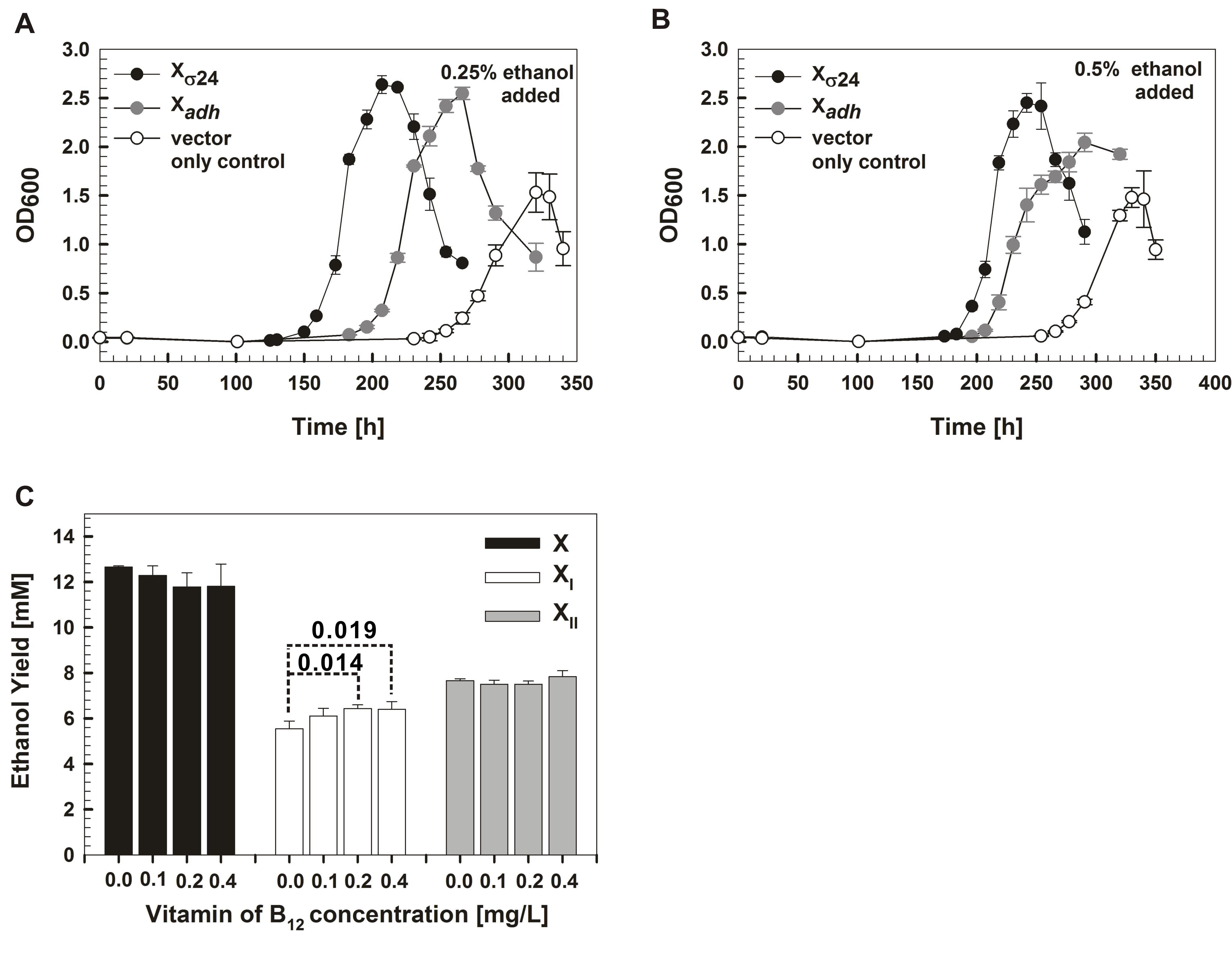

Supplement: Additional file 21 — Genetic and non-genetic approaches that improved both ethanol production and tolerance.A-B) Growth curves of Thermoanaerobacter sp. X514 wild type strains that carried different plasmids (a vector-only control, an adh cluster (Teth5140145-0146) or a ϭ24 cluster (Teth5141847-1848)). Strains were grown at 45°C in QRCM medium with 0.25% and 0.5% added ethanol respectively. C) Fermentation experiments testing effects of vitamin B12 on ethanol production of tolerant mutants in defined medium at 60°C. Vitamin B12 concentrations in defined medium were indicated on x-axis. The means (and standard deviation) for the three biological replicates were shown for each sample. Differences in means were evaluated using one-tailed paired t-test (those with p<0.05 were shown). [file 1754-6834-6-103-S21.tiff]
