# Supplementary material for: Microevolution from shock to adaptation revealed strategies improving ethanol tolerance and production in Thermoanaerobacter
Source: Biotechnol Biofuels. 2013 Jul 22;6:103. doi: 10.1186/1754-6834-6-103 (PMC3751872; doi:10.1186/1754-6834-6-103)
Supplement: Additional file 4 — Statistical analysis of the mutations in X, Xp, XI and XII Genomes. [file 1754-6834-6-103-S4.doc]

**Table S1** Statistical analysis of the mutations in X, Xp, X_I_ and X_II_ genomes.

|  | **X** | **Xp** | **X_I_** | **X_II_** |
| --- | --- | --- | --- | --- |
| Strategy | 2x100bp pair-end | 2x100bp pair-end | 2x76bp pair-end | 1x36bp single-end |
| Total Reads(paired) | 2,500,633 | 21,719,048 | 15,598,762 | 16,013,272 |
| Total Coverage | 95.1% | 96.40% | 96.20% | 95.70% |
| SNPs | 1 | 76 | 20 | 45 |
| SNPs in CDS | 1 | 59 | 17 | 39 |
| SNPs in Inter-gene | 0 | 17 | 3 | 6 |
| SNPs in nonsense mutated CDS | 0 | 14 | 6 | 6 |
| SNPs in missense mutated CDS | 1 | 45 | 11 | 33 |
| Num. of 1 SNP in each CDS | 1 | 36 | 17 | 31 |
| Num. of 2 SNPs in each CDS | 0 | 3 | 0 | 2 |
| Num. of 3 SNPs in each CDS | 0 | 2 | 0 | 0 |
| Num. of 4 SNPs in each CDS | 0 | 0 | 0 | 1 |
| Num. of 11 SNPs in each CDS | 0 | 1 | 0 | 0 |
| changes at genome level | Num. of genes | Num. of genes | Num. of genes | Num. of genes |
| mutated genes with SNPs in CDS | 1 | 42 | 17 | 34 |
| nonsense mutated genes | 0 | 5 | 6 | 6 |
| missense mutated genes (structural proteins) | 0 | 12 | 8 (72.7%**^b^**) | 25 (75.7%**^b^**) |
| missense mutated genes (regulatory factors) | 0 | 4 | 3 (27.2%**^a^**) | 4 (12.1%**^a^**) |
| mutated genes with SNPs in *cis*-element | 0 | 5 | 1 | 4 |

a: Proportion of missense mutations in regulatory factors among total non-sysnonymous mutated genes

b: Proportion of missense mutations in structural proteins among total non-sysnonymous mutated genes
