# Supplementary material for: Microevolution from shock to adaptation revealed strategies improving ethanol tolerance and production in Thermoanaerobacter
Source: Biotechnol Biofuels. 2013 Jul 22;6:103. doi: 10.1186/1754-6834-6-103 (PMC3751872; doi:10.1186/1754-6834-6-103)
Supplement: Additional file 7 — Supplemental Materials. [file 1754-6834-6-103-S7.doc]

**Additional File 7**

Microevolution from shock to adaptation revealed strategies improving ethanol tolerance and production in *Thermoanaerobacter*

**Lu Lin^1^, Yuetong Ji^1^, Qichao Tu^2^, Ranran Huang^1^, Teng Lin^1^, Xiaowei Zeng^1^, Houhui Song^1^, Kun Wang^1^, Yifei Li^1^, Qiu Cui^1^, Zhili He^2^, Jizhong Zhou^2^, and Jian Xu^1,*^**

^1^BioEnergy Genome Center, CAS Key Laboratory of Biofuels and Shandong Key Laboratory of Energy Genetics, Qingdao Institute of BioEnergy and BioProcess Technology, Chinese Academy of Sciences, Qingdao, Shandong, P. R. China

^2^Institute for Environmental Genomics, Department of Microbiology and Plant Biology, University of Oklahoma, Norman, OK, USA

**Running title**: Solvent tolerance and production in thermophiles

***** Corresponding author. Tel.:+ 86 532 8066 2653; fax: +86 532 8066 2654

E-mail address: [xujian@qibebt.ac.cn](mailto:xujian@qibebt.ac.cn) (Jian Xu)

**Part I. The ethanol-“shock” network of the wild type stain revealed novel gene functions.**

Among the 216 ES^+^ nodes, 45 encode hypothetical proteins (**Additional file 6**), representing previously unknown components of ethanol-shock response. An ES^+^-specific hypothetical protein (*teth5141949*) in a dehydratase locus (*teth5141949*-*1953*) was one example. In ES^+^, this locus highly correlated with *teth5141944* and *teth5141954-1955* (microcompartment proteins), *teth5142404* (vitamin B_12_ synthesis) and *teth5141943* (*atr*; converting vitamin B_12_ to coenzyme B_12_) (**Figure 4D**). In the X514 glycobiome underpinning robust ethanol production, *teth5141949* was directly linked to ethanolamine utilization proteins (*teth5141937* and *teth5141946*) and propanediol utilization protein (*teth5141947*). Thus, this gene participated in detoxification under ethanol shock, in contrast to its normal function in robust ethanogenesis.

In addition, in the V-type ATPase centered sub-module of ES^+^, the genes encoding V-type ATPase directly linked to peptidylprolylisomerase (*ppi*; *teth5140594*; involved in protein folding ), stress response genes (*teth5140491*, *teth5141015* (*cas4*) and *teth5141296* (small acid-soluble spore protein, *sasp*) , sporulation gene (*teth5141339*, *yqfD*), antioxidant defense gene (*teth5142241*, *pdxS*) and steroids biosynthesis gene (*teth5140839*, *ygbP*). Noticeably, *ppi*, *sasp* and *pdxS* were present only in ES^+^.

**Part II. Mutated genes in low-ethanol-tolerance community (Xp) and strain (X_I_)**

In DNA replication and repair (COG L), three SNPs, resulting in Ala**^454^**→Thr (68.6%) and Ala**^455^**→Cys (47.7%), were found in the MutL C domain of DNA mismatch repair protein (Teth5141612). MutL, containing an N-terminal ATPase region and a C-terminal dimerization region, is one key component of the DNA repair machinery that corrects replication errors. These mutated sites, located in the N-terminal ATPase, likely perturbed ATP supply and compromised the formation of mismatch DNA signaling complex. Notably, all the SNPs in this protein were located in ATPase domain, indicating the ATPase function might be important to ethanol adaptation of Xp. Another mutation (Thr**^277^**→Ala) was detected in RecA (Teth5141627), a DNA-dependent ATPase. RecA protein catalyses an ATP-dependent DNA strand-exchange reaction that is the central step in the repair of dsDNA breaks by homologous recombination . Therefore, these SNPs might compromise the DNA repair mechanism and thus accelerate genome mutation.

In transcription regulation (COG K), one SNP (Asp**^961^**→Gly) was found in domain 6 of the RNA polymerase subunit Rpb2 (Teth5140859). In the RNA Pol II transcription elongation complex, Rpb2 binds the complex formed by the nascent RNA strand and the template DNA strand .

In protein translation (COG J), a Val**^102^**→Ala was found in ribosomal protein S12 (Teth5140862), which is involved in the translation initiation step and an Ala**^107^**→Val was identified in ribosomal protein L16, which is known to bind directly the 23S rRNA. These SNPs suggested ethanol tolerance might involve protein synthesis.

In X_I_, one appeared beneficial mutations lay in electron transport complex I (Teth5140079; Ala**^270^**→Pro) In COG C, which likely resulted in reduced ATP production (Electron transfer build the [electrochemical potential](http://en.wikipedia.org/wiki/Electrochemical_potential) for ATP production ), consistent with inhibition of energy-demanding processes in X_I_ (e.g., slower growth, **Additional file 2A**)). The other one (Gly**^100^**→Asp) was detected in TrkH family potassium uptake protein (Teth5140140) In COG P involved in active sodium up-take. Sodium transport is implicated in the maintenances of pH homeostasis, osmotic pressure and metabolism balance.

**Part III. *A priori* ethanol stress rewired additional aspects of the gene networks.**

*A priori* ethanol stress left striking footprints in the genetic underpinning of X_I_-0%. The expression levels of genes involved in vitamin B biosynthesis, stress response pathways, nitrogen- metabolism and cell wall/membrane metabolism were also significantly changed (X-0% as the baseline).

(i) Vitamin B biosynthesis. In X_I_-0%, riboflavin synthesis (*teth5140021-0022*, vitamin B_2_), pantothenate and CoA biosynthesis (*teth5140426-0428*, vitamin B_5_) and thamine synthese (*teth5140565-0569*, vitamin B_1_) were upregulated. Vitamin B_2_ plays a key role in energy metabolism, fatty acid synthesis, [carbohydrates](http://en.wikipedia.org/wiki/Carbohydrate) metabolism, and protein synthesis . B_5_ is involved in cell wall and membrane biosynthesis , whereas B_1_ contributes to cellular resistance to divalent metal ions, antibiotics and H_2_O_2_ .

(ii) Stress responses. Even in the absence of ethanol, several genes were induced in X_I_-0% (**Additional file 15A**). In X_I_-0%, defense mechanism (COG V) and posttranslational modification and chaperones genes (COG O) were up-regulated, including peptidoglycan binding domain-containing protein (*teth5140954*), restriction modification system (*teth5141221*-*1222*), and protease/peptidase (*teth5141034* and *teth5142047-2048*).

(iii) Nitrogen metabolism. Biosynthesis genes for histidine, leucine, tryptophan, and methionine were upregulated in X_I_-0% (**Additional file 15A**), explaining its higher biomass than X in the absence of ethanol (**Additional file 2A**). However, ethanolamine utilization proteins (*teth5141943-1946*), whose expression level positively correlates with ethanol production in X514 glycobiome , were down-regulated, consistent with the lower ethanol productivity .

(iv) Cell wall/membrane metabolism and related transporters. A *priori* ethanol stress inhibited cell wall/membrane metabolism and related transporters in X_I_. Cell wall hydrolyase/autolysin (*teth5140925-0926*) was inhibited in X_I_-0% (**Additional file 15A**), which hydrolyzes the shape-maintaining and stress-bearing peptidoglycan layer of cell wall and is involved in cell separation, motility and cell lysis . The lower activity might decrease cell permeability of X_I_. Peptidoglycan biosynthesis genes (*teth5142008-2017*) were also inhibited (**Additional file 15A**), whose products give physical strength to cell wall structure.

Besides cell membrane metabolism, several transport system genes were down-regulated, including carbohydrate transport systems (fructose-, glucose-, mannitol- and cellobiose-specific PTS systems (*teth5140824*, *teth5140412-0413*, *teth5140268* and *teth5140239*), sodium pump decarboxylase (*teth5141850-1851*), dipeptide ABC transporters (*teth5141792-1796* and *teth5141852-1853*) and ion ABC transporters (*teth5140297-0326*, and *teth5141932-1934*) (**Additional file 15A**). Thus the across-membrane transport decreased in low-tolerance mutant.

**Part IV. Additional mutations that were shared between Xp and X_II_**

In both Xp and X_II_, DeoR family transcriptional factor (Teth5141305), a central regulator of glycolysis, harbored an Asn**^133^**-to-Ser mutation in the C-terminal effector-binding domain (**Additional file 17A**). DeoR family TF, as a repressor, negatively regulates the phosphorylation of intermediates in sugar metabolic pathways . When ligands (carbohydrate intermediates of glycolysis, e.g. fructose-1, 6-bisphosphate) bind to DeoR, this repression is abolished . As the ligands are structurally distinct, wild-type DeoR lacks specific sugar-binding motifs. Thus, ligand binding occurs at the cost of binding energy . We inferred this mutation might facilitate binding of ligand to DeoR in X_II_ to reduce cellular energy consumption under stress, consistent with the reduced cellular energy consumption under stress . Other shared mutations were in NusG anti-termination factor (Pro**^34^**→Ser in NusG domain, Teth5142239), integral membrane sensor signal transduction histidine kinase (Ser**^431^**→Arg (Xp) and Glu**^394^**→Thr (X_II_) in the ATPase domain, Teth5142217) and the upstreams of the *teth5142105* and *teth5141994* respectively (**Additional file 11**).

In addition, X_II_ harbored additional SNPs that were absent in both Xp and X_I_. They were mostly in two categories: ribose metabolism and cell membrane metabolism. First, one SNP (Thr**^94^**→Ala in Teth5140168) was located between HTH and SIS (Sugar Isomerase) domains in an RpiR family transcriptional regulator that regulates the ribose catabolism . A Gly**^617^**→Arg mutation was found in the PTS system fructose IIA domain of ϭ^54^ factor interaction domain-containing protein (Teth5140261). These two specific mutated TFs, together with the mutated DeoR TF and AdhE (in X_II_), suggested their key roles in ethanol adaptation. Second, a G→A substitution was detected at 12bp upstream of Teth5142105, which is involved in cell wall synthesis. A Thr**^341^**→Pro was identified in the SIS domain of a glucosamine-fructose-6-phosphate aminotransferase (Teth5140950) which synthesizes glucosamine-6-phosphate, a precursor to peptidoglycan and cell wall lipopolysaccharides (LPS) . Another SNP (Val**^237^**→Ile) was located in the peptidoglycan binding domain (present at N or C terminus of a variety of bacterial cell wall degrading enzymes ) of Teth5140925. Thus the reshaped membrane metabolism in X_II_ contributed to enhance ethanol tolerance.

**Part V. Additional** **transcriptomic features of X_II_-0% in comparison to X-0%**

*A priori* ethanol stress also left striking footprints in the genetic underpinning of X_II_-0%. The expression levels of genes involved in stress response pathways, nitrogen- metabolism and cell wall/membrane metabolism were also significantly changed (X-0% as the baseline).

(i) Stress responses. Even in the absence of ethanol, several genes in stress response pathways were induced in X_II_-0% (**Additional file 15B**). Defense mechanism (COG V) and posttranslational modification and chaperones genes (COG O) were up-regulated, including restriction modification system (*teth5141221*-*1222*) and cytochrome *c* biogenesis protein (*teth5141434*). In addition, efflux pump systems were specifically employed (up-regulated) (**Additional file 15B**). A TetR family TF (*teth5141173*) was induced, which modulates multidrug efflux pumps, antibiotics biosynthesis and genes responsive to osmotic stress and toxic chemicals . Also induced were major facilitator transport systems (*teth5141765-1766*), which transport small solutes in response to chemiosmotic ion gradients to maintain ATP generation , and sodium:neurotransmitter symporter (*teth5141105*) that provides osmoprotection via transporting proline, glycine, choline and betaine that protect cell from osmotic stress .

Moreover, oxidoreductase stress response was observed, as oxidoreductase genes were upregulated in X_II_-0%, such as glutamate synthase (*teth5140502-0503*), aldoreductase (*teth5140625*). Thus various stress response pathways were specifically turned on in X_II_-0%, explaining its higher ethanol tolerance.

However, the induction of molecular chaperons e.g. HSPs) were absent under either shock or stress. Molecular chaperons, participating in protein folding and protecting cells from stresses, were induced as one of the most prominent and universal response to ethanol stress in mesophiles (e.g., *Clostridium acetobutylicum*, *E.coli*  and *S. cerevisiae* . In fact, under normal conditions (50mM glucose in defined medium at 60^o^C for X514; 28mM glucose in CGM medium at 35^o^C for *C. acetobutylicum* ), thermophiles maintained high transcriptional levels of *hsp*s: *hsp20* was among the top 0.6% of genes based on transcript abundance (the 14^th^ highest transcribed gene) in X514 yet was among the lowest 54.6% (ranking 2099^th^ in transcript level) in *C. acetobutylicum* (the latter was consistent with the current notion of the very-low presence of molecular chaperones in mesophiles ). Therefore, HSPs seems sustain their high levels in thermophiles in the absence of stress.

(ii) Nitrogen metabolism. Biosynthesis of arginine (*teth5140661-0662* and *teth5140664*) and glutamate (*teth5140651-0652*) was repressed, consistent with its slower growth than X-0% (**Additional file 2A** and **Additional file 15B**).

(iii) Cell wall/membrane metabolism and related transporters. Repressed cell wall hydrolyase/autolysin (*teth5140925-0926*) and peptidoglycan biosynthesis genes (*teth5142015-2017*) in X_I_-0% were also observed in X_II_-0% (**Additional file 15B**). Furthermore, operon structure appeared to be modulated along tolerance development. One example was *teth5140597*-*0601*. In X-0%, the genes were transcribed in one single polycistron, i.e, as one operon (**Additional file 18A**). However in X_II_-0%, their transcription was split into three polycistrons: *teth5140597*, *teth5140598* and *teth5140599*-*0561* (**Additional file 18B**). Abundance of *teth5140597* transcripts (encoding a hypothetical protein) was not significantly changed. That of *teth5140598* (encoding peptidoglycan-binding LysM involved in cell wall degradation) was down-regulated in X_II_-0%. Those of *teth5140599*-*0601*, involved in terpenoid, molybdopterin-guanine dinucleotide biosynthesis and gluconate metabolism regulation, were not significantly changed. Therefore, *a priori* ethanol stress left striking footprints on their regulatory mode and cellular metabolisms, even in the absence of contemporary exogenous ethanol.

**Part VI. Genes that were transcriptionally repressed in X_II_-6% when compared to X_II_-2%**

The 725 downregulated genes were mainly those involved in transport and metabolism of carbohydrate, ion and amino acids, energy metabolism and DNA replication and translation. Several were known to play pivotal roles in ethanol production: *adh*s (*teth5140241*, *teth5140653-0654* and *teth5141935*), *aldh* (*teth5141942*) and B_12_ biosynthesis genes (*teth5140323-0327*), whose lower expression and the undetectable ethanol yields in X_II_-6% (**Additional file 2B**) were a sharp contradiction to the networks of robust ethanol production (where these genes were actively expressed and positively correlated with ethanol yield ).

**Part VII. Improving ethanol titer of the low-tolerance mutant via vitamin B12 supplementation**

The microevolution model suggested a role of B_12_ biosynthesis in ethanol-shock response, as the underlying genes existed specifically in ES^+^ (but not in ES^-^; **Figure 4D**). Moreover, it might contribute to ethanol production in the “high-tolerance” phase, as from X_I_ to X_II_, transcript level of the genes increased at least 2.3 folds. Such an expression pattern correlated with the 55% higher ethanol production in X_II_ than X_I_ (**Additional file 2B**) and was consistent with our previous report that B_12_ biosynthesis contributed to ethanolgenesis in *Thermoanaerobacter* . To further test and potentially exploit the effects, X, X_I_ and X_II_ were grown respectively on glucose with supplemented exogenous B_12_ (0, 0.1, 0.2 and 0.4 µg/ml) in defined medium at 60^o^C. Ethanol production in X and X_II_ were largely independent of B_12_ concentration, however for X_I_, it increased by 16% (*p* = 0.014; **Additional file 21C**).

**References**

1. Lin L, Song H, Tu Q, Qin Y, Zhou A, Liu W, He Z, Zhou J, Xu J: **The *Thermoanaerobacter* Glycobiome Reveals Mechanisms of Pentose and Hexose Co-Utilization in Bacteria.** *PLoS Genet* 2011, **7 (10):**e1002318.

2. Fischer G, Schmid FX: **The mechanism of protein folding. Implications of in vitro refolding models for de novo protein folding and translocation in the cell.** *Biochemistry* 1990, **29:**2205-2212.

3. Makarova KS, Aravind L, Grishin NV, Rogozin IB, Koonin EV: **A DNA repair system specific for thermophilic Archaea and bacteria predicted by genomic context analysis.** *Nucleic Acids Res* 2002, **30:**482-496.

4. Guarne A, Ramon-Maiques S, Wolff EM, Ghirlando R, Hu X, Miller JH, Yang W: **Structure of the MutL C-terminal domain: a model of intact MutL and its roles in mismatch repair.** *EMBO J* 2004, **23:**4134-4145.

5. Savir Y, Tlusty T: **RecA-Mediated Homology Search as a Nearly Optimal Signal Detection System.** *Molecular Cell* 2010, **40:**388-396.

6. Gnatt AL, Cramer P, Fu J, Bushnell DA, Kornberg RD: **Structural basis of transcription: an RNA polymerase II elongation complex at 3.3 A resolution.** *Science* 2001, **292:**1876-1882.

7. Turner PC, Mclennan AG, Bates AD, White MRH: *Instant Notes in Molecular Biology.* 3rd edn. Liverpool, UK: University of Liverpool; 2001.

8. Bond DR, Marsili E, Baron DB, Shikhare ID, Coursolle D, Gralnick JA: ***Shewanella* secretes flavins that mediate extracellular electron transfer.** *Proc Natl Acad Sci USA* 2008, **105:**3968-3973.

9. Toennies G, Das DN, Feng F: **Pantothenate and coenzyme A in bacterial growth.** *J Bacteriol* 1966, **92:**707-713.

10. Hustavova H, Havranekova D: **The role of thiamine in *Yersinia kristensenii* resistance to antibiotics and heavy metals.** *Folia Microbiol (Praha)* 1997, **42:**569-573.

11. Navarre WW, Schneewind O: **Surface proteins of gram-positive bacteria and mechanisms of their targeting to the cell wall envelope.** *Microbiol Mol Biol Rev* 1999, **63:**174-229.

12. Rezacova P, Kozisek M, Moy SF, Sieglova I, Joachimiak A, Machius M, Otwinowski Z: **Crystal structures of the effector-binding domain of repressor Central glycolytic gene Regulator from *Bacillus subtilis* reveal ligand-induced structural changes upon binding of several glycolytic intermediates.** *Mol Microbiol* 2008, **69:**895-910.

13. Stanley D, Bandara A, Fraser S, Chambers PJ, Stanley GA: **The ethanol stress response and ethanol tolerance of *Saccharomyces cerevisiae*.** *J Appl Microbiol* 2010, **109:**13-24.

14. Sorensen KI, Hove-Jensen B: **Ribose catabolism of *Escherichia coli*: characterization of the *rpiB* gene encoding ribose phosphate isomerase B and of the *rpiR* gene, which is involved in regulation of *rpiB* expression.** *J Bacteriol* 1996, **178:**1003-1011.

15. Kalamorz F, Reichenbach B, Marz W, Rak B, Gorke B: **Feedback control of glucosamine-6-phosphate synthase GlmS expression depends on the small RNA GlmZ and involves the novel protein YhbJ in *Escherichia coli*.** *Mol Microbiol* 2007, **65:**1518-1533.

16. Bateman A, Bycroft M: **The structure of a LysM domain from *E. coli* membrane-bound lytic murein transglycosylase D (MltD).** *J Mol Biol* 2000, **299:**1113-1119.

17. Ramos JL, Martinez-Bueno M, Molina-Henares AJ, Teran W, Watanabe K, Zhang X, Gallegos MT, Brennan R, Tobes R: **The TetR family of transcriptional repressors.** *Microbiol Mol Biol Rev* 2005, **69:**326-356.

18. Pao SS, Paulsen IT, Saier MH, Jr.: **Major facilitator superfamily.** *Microbiol Mol Biol Rev* 1998, **62:**1-34.

19. He Z, Zhou A, Baidoo E, He Q, Joachimiak MP, Benke P, Phan R, Mukhopadhyay A, Hemme CL, Huang K, et al: **Global transcriptional, physiological, and metabolite analyses of the responses of *Desulfovibrio vulgaris* hildenborough to salt adaptation.** *Appl Environ Microbiol* 2009, **76:**1574-1586.

20. Tomas CA, Beamish J, Papoutsakis ET: **Transcriptional analysis of butanol stress and tolerance in *Clostridium acetobutylicum*.** *J Bacteriol* 2004, **186:**2006-2018.

21. Goodarzi H, Bennett BD, Amini S, Reaves ML, Hottes AK, Rabinowitz JD, Tavazoie S: **Regulatory and metabolic rewiring during laboratory evolution of ethanol tolerance in *E. coli*.** *Mol Syst Biol* 2010, **6:**378.

22. Ma M, Liu ZL: **Mechanisms of ethanol tolerance in *Saccharomyces cerevisiae*.** *Appl Microbiol Biotechnol* 2010, **87:**829-845.

23. Servinsky MD, Kiel JT, Dupuy NF, Sund CJ: **Transcriptional analysis of differential carbohydrate utilization by *Clostridium acetobutylicum*.** *Microbiology* 2010, **156:**3478-3491.

24. Ambily Nath I.V., LokaBharathi PA: **Diversity in transcripts and translational pattern of stress proteins in marine extremophiles.** *Extremophiles* 2011, **15:**129-153.

25. Lin L, Song H, Tu Q, Qin Y, Zhou A, Liu W, He Z, Zhou J, Xu J: **The *Thermoanaerobacter* glycobiome reveals mechanisms of pentose and hexose co-utilization in bacteria.** *PLoS Genet* 2011, **7:**e1002318.
