# Supplementary material for: Microevolution from shock to adaptation revealed strategies improving ethanol tolerance and production in Thermoanaerobacter
Source: Biotechnol Biofuels. 2013 Jul 22;6:103. doi: 10.1186/1754-6834-6-103 (PMC3751872; doi:10.1186/1754-6834-6-103)
Supplement: Additional file 12 — Experimental validation of SNPs in XI and XII. A) The primer sequences used for PCR and then Sanger sequencing. B) The SNPs validated by Sanger sequencing. [file 1754-6834-6-103-S12.doc]

**Table S4 Experimental validation of SNPs in X_I_ and X_II_. A): The primer sequences used for PCR and then Sanger sequencing. B): The SNPs validated by Sanger sequencing.**

| **A** | | | |
| --- | --- | --- | --- |
| **Gene ID** | **Forward primer** | **Reverse primer** | **Predicted function of selected protein** |
| Teth5140216 | TGAGTTCTTCCATCAAAGAC | GCTCCCGCTGTAATGATT | Lactate dehydrogenase |
| Teth5140089 | GAGATTAGAGACTTCACCTACA | AAGCAGTAGCAAGGATAGTT | transcription termination factor Rho |
| Teth5140284 | GGACCTTCGACAATACCAA | GCCGTTATGACCCTTAGG | LysR family transcriptional regulator |
| Teth5141376 | TCAAACAGGAGAGAAGTGG | ATCGCTCATCTGCATAGTA | CRP/FNR family transcriptional regulator |
| Teth5142105 | AATGGTCTTCCTGATGACAA | ATTCGGCTGCAATGAGAG | Serine-type D-Ala-D-Ala carboxypeptidase |
| Teth5140627 | CGGCTGCTTTGGGATATTCA | CTGTAGGTGGTGGTTCTG | bifunctional acetaldehyde-CoA/alcohol dehydrogenase |
| Teth5141994 | AAACGATAGCCTTTCCAAAC | AGGTCAGGAAGAAGCAGAA | phosphate ABC transporter, inner membrane subunit PstC |
| Teth5142105 | ATGGCATACTGTTGTCTCTT | CTACGCTTCCACCAATGT | Serine-type D-Ala-D-Ala carboxypeptidase |

| **B** | | | | | |
| --- | --- | --- | --- | --- | --- |
| **Strain** | **Gene ID** | **location** | **Reference** | **SNP** | **SNP vs Reference**^a^ |
| X_I_ | Teth5140216 | 225986-225987 | GA | GTA | 10 (T) : 0 (-) |
| X_I_ | Teth5140089 | 88991 | A | G | 6(G) vs 4(A) |
| X_I_ | Teth5140284 | 304240 | A | G | 6(G) vs 4(A) |
| X_I_ | Teth5141376 | 1417599 | G | A | 10(A) vs 0(G) |
| X_I_ | Teth5142105 | 2114002 | G | T | 10(T) vs 0(G) |
| X_II_ | Teth5140216 | 225986-225987 | GA | GTA | 10 (T) : 0 (-) |
| X_II_ | Teth5140627 | 652702 | T | C | 10(C) vs 0(T) |
| X_II_ | Teth5140627 | 653152 | A | T | 10(T) vs 0(A) |
| X_II_ | Teth5142105 | 2114002 | G | T | 10(T) vs 0(G) |
| X_II_ | Teth5142105 | 2113303 | G | A | 10(A) vs 0(G) |
| X_II_ | Teth5141994 | 2004893 | ATT | A-T | 10(-) vs 0(T) |

a: Number of clones detected with different bases at SNP position
